# iGlucoSnFR2: A genetically encoded fluorescent sensor for measuring intracellular or extracellular glucose in vivo in mouse brain

**DOI:** 10.1126/sciadv.adz3889

**Published:** 2025-11-12

**Authors:** Jonathan S. Marvin, Philipp Mächler, Chengbo Meng, Tayfun Ates, Ronak H. Patel, Raghabendra Adhikari, Monika A. Makurath, Zaneta Ku, Daniel Feliciano, Deniz Atasoy, Guohong Cui, David Kleinfeld, Timothy A. Brown

**Affiliations:** ^1^ Howard Hughes Medical Institute, Janelia Research Campus, Ashburn, VA, USA.; ^2^ Department of Physics, University of California at San Diego, La Jolla, CA, USA.; ^3^ In Vivo Neurobiology Group, Neurobiology Laboratory, National Institute of Environmental Health Sciences, National Institutes of Health, Research Triangle Park, NC, USA.; ^4^ Department of Neuroscience and Pharmacology, University of Iowa Carver College of Medicine, Iowa City, IA, USA.; ^5^ Department of Neurobiology, University of California at San Diego, La Jolla, CA, USA.

## Abstract

Continuous glucose monitors have proven invaluable for monitoring blood glucose levels for diabetics, but they are of limited use for observing glucose dynamics at the cellular (or subcellular) level. We have developed a second generation, genetically encoded intensity-based glucose sensing fluorescent reporter (iGlucoSnFR2). We show that when it is targeted to the cytosol, it reports intracellular glucose consumption and gluconeogenesis in cell culture, along with efflux from the endoplasmic reticulum. It outperforms the original iGlucoSnFR in vivo when observed by fiber photometry in mouse brain and reports transient increase in glucose concentration when stimulated by noradrenaline or electrical stimulation. Last, we demonstrate that membrane localized iGlucoSnFR2 can be calibrated in vivo to indicate absolute changes in extracellular glucose concentration in awake mice. We anticipate iGlucoSnFR2 facilitating previously unobservable measurements of glucose dynamics with high spatial and temporal resolution in living mammals and other experimental organisms.

## Teaser

Genetically encoded fluorescent sensor reports extracellular or intracellular glucose fluctuations in mouse brains in vivo.

## INTRODUCTION

Monitoring glucose dynamics is crucial for understanding its role in normal physiology and pathological conditions. Glucose homeostasis is maintained by storing glucose as glycogen in the the liver and muscles (*1*) and mobilizing it to energetically demanding tissues when needed. Glycogen use is primarily regulated by the secretion of insulin and glucagon from the pancreas (*2*). Glucose serves as the primary energy substrate for the brain (*3*), with its availability and metabolism intricately linked to neuronal activity, synaptic plasticity, and overall cognitive function. Dysregulation of glucose metabolism is implicated in a range of neurological disorders including Alzheimer’s disease (*4*) and epilepsy (*5*), along with metabolic diseases affecting key glucose-regulating tissues such as the liver (e.g., type 2 diabetes and obesity). While blood glucose remains relatively constant, intracellular glucose concentrations—particularly within metabolically active cells such as neurons and hepatocytes or subcellular compartments such as the endoplasmic reticulum (ER)—can fluctuate and reflect important metabolic states in health and disease. Therefore, tools that enable precise, real-time measurement of glucose in energy-demanding or metabolic regulatory tissues and subcompartments throughout the body are indispensable for advancing both basic neuroscience and translational research.

Historically, enzyme-based sensors or microdialysis coupled with liquid chromatography–mass spectrometry, have been used to measure the concentration of scientifically interesting analytes. However, these techniques are limited in their spatial and temporal resolution. Genetically encoded fluorescent sensors have emerged as powerful tools for monitoring neurotransmitters (*6*) and metabolites (*7*) with high spatial and temporal resolution. These sensors, mostly based on fluorescent proteins (FPs), allow for noninvasive, cell type–specific measurements and can be targeted to subcellular compartments, enabling researchers to uncover the complex, localized dynamics of the analyte of interest.

Multiple genetically encoded fluorescent sensors for glucose have been developed from the glucose/galactose binding protein of *Escherichia coli* (MglB) and have been reviewed recently (*8*, *9*). One of the first, FLIP-glu (fluorescent indicator protein for glucose), was a Förster resonance energy transfer (FRET) sensor based on MglB coupled to cyan and yellow FPs (*10*, *11*). While FLIP-glu has been cited more than 300 times and has been used in hippocampal slice preparations (*12*, *13*), its use has mostly been limited to cell culture, likely because of its optical parameters (excitation with 435-nm light matches the absorption peak of hemoglobin), limited dynamic range (30% ratio change), and issues arising from recombination events during adeno-associated virus (AAV) production for in vivo expression (*14*). Green Glifon (*15*) and Red Glifon (*16*) have been developed using split citrine (a yellow fluorescent protein) and split mApple (a red FP), respectively, providing single wavelength genetically encoded glucose sensors, with about fivefold changes in fluorescence intensity upon binding glucose, while these could be useful for in vivo imaging that has yet to be demonstrated. One factor that might impede their implementation in vivo is the relatively slow kinetics that occur when using split FPs instead of circularly permuted FPs, as was observed with a similarly developed adenosine triphosphate (ATP) sensor, MaLion-G (*17*).

An intensity-based glucose-sensing fluorescent reporter, iGlucoSnFR (referred to here as iGlucoSnFR1), was developed from the glucose binding protein of *Thermus thermophilus* and circularly permuted green fluorescent protein (cpGFP) (*18*). This glucose binding protein has a different tertiary topology than MglB; it is more structurally homologous to maltose-binding protein (*19*), from which we developed a maltose sensor (*20*). This protein has the advantages of being about 70 amino acids longer than MglB, with more structural elements that can be identified as conformationally coupled to ligand binding. It is also thermostable, making it potentially more structurally tolerant of mutations and insertion of a circularly permuted FP. The iGlucoSnFR1 sensor was used to report changes in glucose concentration not only in cell culture but also in vivo in *Drosophila* and zebrafish. However, its utility in vivo in mammals was not demonstrated, but a lifetime-sensing derivative, iGlucoSnFR-TS, has been used in vivo in mouse visual cortex (*21*).

Here, we describe the development and characterization of a second-generation sensor, iGlucoSnFR2. This improved version replaces circularly permuted eGFP of iGlucoSnFR1 with circularly permuted SuperFolder GFP (cpSFGFP) and shows substantial improvements. It has a higher maximum change in fluorescence (∆*F*/*F*) and has a more appropriately tuned glucose-binding affinity with a dissociation constant (*K*_d_) around 500 μM, as the concentration of glucose in the interstitial space of rat brain is much lower than blood glucose levels (*22*). Last, it has vastly improved subcellular localization efficiency. These modifications enable more accurate and reliable monitoring of glucose dynamics across a broad spatial range (from subcellular to organ-wide, including the brain) with high temporal resolution, paving the way for new insights into the metabolic underpinnings of brain function and dysfunction.

## RESULTS

### Protein engineering and in vitro characterization

**Fig. 1.**
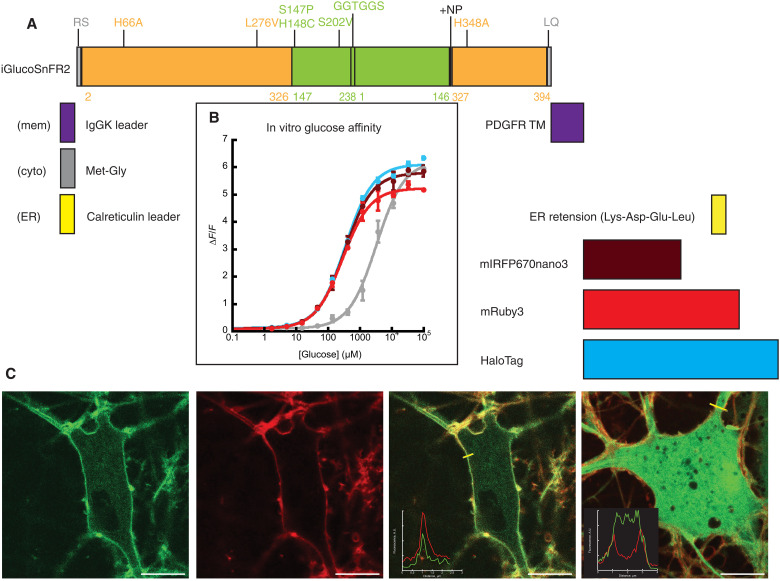
Design and initial characterization of iGlucoSnFR2. (**A**) Linear map of iGlucoSnFR2 with mutations to the junctions between binding protein and cpSFGFP (junction 1 = S147P, H148C; junction 2 = insertion of NP) along with chromophore proximal mutation GFP.S202V and affinity modulation mutations (H66A, L276V, and H348A) within the glucose-binding protein itself. Amino acids Arg-Ser (RS) and Leu-Gln (LQ) at the beginning and end of the sensor encode Bgl II and Pst I restriction sites for subcloning into mammalian expression vectors. Subcellular targeting to the cytosol (cyto), plasma membrane (mem), or ER was accomplished by inclusion of sequences flanking the sensor. C-terminal normalization tags were also included as diagramed. (**B**) Affinity curves of iGlucoSnFR2 with different C-terminal tags. Color scheme matches (A), and gray is untagged iGlucoSnFR2. Glucose affinity for tagged proteins is 300 μM and 3 mM for untagged. Error bars are SD of three technical replicates. (**C**) Confocal imaging (60×) of iGlucoSnFR2 targeted to the plasma membrane (three left images) or cytosol (right) of neurons. Costained with the Nile Red–based membrane dye NR12S. Scale bars, 10 μm. A.U., arbitrary unit; PDGFR, platelet-derived growth factor receptor; TM, transmembrane.

The glucose sensor iGlucoSnFR1 was developed in 2012 as one of our original proof-of-principle sensors (*23*), but its use in different biological models was not published until 2021 (*18*). In the intervening years, we have developed other intensity-based fluorescent sensors and improved design and screening techniques. By retrospectively analyzing iGlucoSnFR1, we realized that we could make a better sensor with some straightforward changes. First, we observed that iGlucoSnFR1 did not translocate to the plasma membrane when targeted there by an immunoglobulin G kappa light chain secretion signal and a C-terminal platelet-derived growth factor receptor transmembrane anchor (fig. S1). SFGFP is a variant of GFP that was selected to fold well even when fused to poorly folded partner proteins (*24*). We have learned that replacing circularly permuted enhanced GFP (cpeGFP) with cpSFGFP helps other sensors translocate to the membrane, presumably by improving folding in the ER. We had anecdotal evidence that reoptimizing the codon usage from the native bacterial thermophilic sequence to one that better matches the codon usage of prevalent experimental animal models (mouse) helps expression levels. We also learned that making direct fusions of the binding protein to cpSFGFP followed by optimization of the sequence at their juncture results in sensors with higher fluorescence changes than when extra residues (“linkers”) are inserted between the two proteins and that other residues near the chromophore of GFP play an important role in modulating fluorescence in cpFP-based sensors. Thus, we embarked on reengineering iGlucoSnFR1 by replacing cpeGFP with cpSFGFP using a mouse-codon–optimized version of the glucose binding protein, performing a fresh screen of mutants of the binding protein and FP junctions and screening mutants of a few select positions near the chromophore of SFGFP. We identified a variant with Junction1-PC, Junction2-NP, and SFGFP.S202V that has a ∆*F*/*F* near 5 and a *K*_d_ for glucose of ~500 μM; we call it iGlucoSnFR2 (Fig. 1).

Experiments performed over short timeframes with stable focus are ideal settings for intensity-based sensors. For other experiments, it is sometimes valuable to have a method for distinguishing fluorescence changes that occur from changes in analyte concentration from those that result from changes in protein expression or focus. One option for normalization is to have a sensor based on two diametrically opposed fluorescence intensity changes (i.e., FRET-based sensors). Another option is to add a nonresponsive reference channel. We have developed iGlucoSnFR2 using the latter approach.

In the original work, we observed that fusion of the red FP mRuby2 decreased the maximum fluorescence change ratio (∆*F*/*F*) of iGlucoSnFR1. To resolve this issue, we made multiple iGlucoSnFR2-fusion proteins and characterized them in vitro. In addition to testing naked iGlucoSnFR2, we made variants with C-terminal normalization fusions to the FPs mRuby3 [excitation/emission (Ex/Em) of 558/580], mIRFP670nano3 (Ex/Em of 650/670), and HaloTag (*25*), a protein that has been engineered to form covalent bonds with synthetic small molecules [such the Janelia Fluor series of fluorescent dyes (*26*)]. These FPs were chosen because they can be spectrally separated from the GFP-based sensor using commercially available Texas Red or Cy5 filters. These fusions allow normalization of the GFP sensor signal to a nonresponsive red/far-red signal. Figure 1 shows how those fusions proteins affect both maximum ∆*F*/*F* and affinity. The sensor is specific for glucose, not binding phosphorylated glucose decoys nor related sugars. Its fluorescence is sensitive to pH, as are all cpGFP-based sensors, but pH affects the bound and unbound states equally. The spectra, affinity for relevant decoy ligands, pH dependence, and kinetics of iGlucoSnFR2 are shown in fig. S1. We also reverted a binding site residue back to its original identity to create a higher affinity variant (H348H). Combined, these versions provide opportunities to query changes in glucose concentrations under a variety of conditions and with different imaging setups.

### Validation of iGlucoSnFR2 in cell culture—Glucose depletion

We wanted to demonstrate that iGlucoSnFR2 could be used to observe cellular changes in glucose concentrations in different regimes: depletion, repletion, consumption, and synthesis. To observe depletion, we transiently transfected HeLa cells with plasmids expressing cytosolically targeted cpSFGFP-HaloTag, iGlucoSnFR1-HaloTag, or iGlucoSnFR2-HaloTag; incubated them with HaloTagLigand-JFX650; and imaged them in a multi-well imager. Cells were equilibrated with buffer containing 1, 2, 5, or 10 mM glucose. At the treatment point, we added an equal volume of buffer (containing the same concentration glucose as the equilibration buffer) plus Glutor at a final concentration of 10 μM. Glutor inhibits bidirectional glucose transporters GLUT-1, GLUT-2, and GLUT-3 (*27*). As expected, the cpSFGFP control does not respond but for a transient increase in fluorescence when the perturbation of the media is performed (fig. S2A). This is an artifact we have observed previously with the Cytation imager (*28*). iGlucoSnFR1 reports the inhibition of glucose transport as expected, with greater drops in fluorescence occurring with higher concentrations of glucose (fig. S2B). iGlucoSnFR2 shows a greater drop in fluorescence (fig. S2C) than iGlucoSnFR1, which is a result of both higher ∆*F*/*F* and sensor affinity. Cytosolic iGlucoSnFR2 is saturated at 5 mM external glucose; increasing the external concentration of glucose to 10 mM does not increase the GFP:JFX650 ratio. We repeated the experiment with three additional immortalized cell lines: human embryonic kidney–293, U2OS (osteosarcoma), and COS-7 (monkey kidney). All three had GFP/JFX650 ratios similar to the HeLa (cervical cancer) cells and responded similarly to Glutor treatment (fig. S3).

**Fig. 2.**
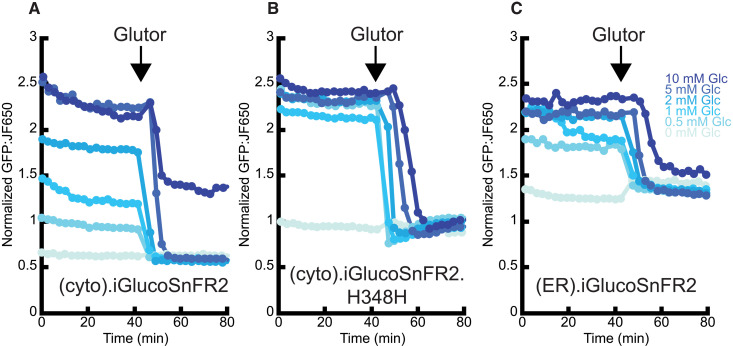
iGlucoSnFR2 reports effects of GLUT inhibition on intracellular glucose in HeLa cells. (**A**) (cyto).iGlucoSnFR2, (**B**) (cyto).iGlucoSnFR2.H348H, and (**C**) (ER).iGlucoSnFR2 each with HaloTag-JFX650. Equilibration glucose (Glc) concentration indicated by increasing blue hue: 0, 0.5, 1, 2, 5, or 10 mM glucose. Arrow indicates time of treatment with 10 μM Glutor. Each trace is an average of the green/far-red fluorescence ratio of the cells within the field of view (FOV). The average number of cells per FOV is 24.1, with a range of 7 to 48.

We also made a higher affinity (*K*_d_ ~ 3 μM) variant of iGlucoSnFR2 with a binding pocket residue reverted to its wild-type identity, H348H. We repeated the Glutor-treatment experiment in HeLa cells with cytosolic iGlucoSnFR2, iGlucoSnFR2.H348H, or ER-targeted iGlucoSnFR2 (Fig. 2). Treatment with Glutor causes a drop in cytosolic iGlucoSnFR2 fluorescence as expected. The H348H variant appeared saturated at 1 mM external glucose and shows a lag between the start of Glutor treatment and the drop in fluorescence at higher equilibration concentrations of glucose, demonstrating its insensitivity to high concentrations glucose. However, once glucose is consumed and not replaced by transport, the H348H variant shows a slow increase in cytosolic glucose. This implies that inhibition of glucose transport from the external buffer into the cell results in release of glucose from intracellular storage spaces into the cytosol after the bulk of it is consumed. Since this phenomenon was only observed with the high affinity variant, the glucose released into the cytosol must be at concentration lower than the *K*_d_ of the normal sensor (<500 μM) but higher than the *K*_d_ of the high affinity sensor (>3 μM). We repeated the experiment using a perfusion apparatus and measured changes in cytosolic glucose of individual cells as buffer was switched from buffer containing 10 or 50 μM glucose to zero glucose and back (fig. S4A). The fluorescence of the sensor in cells dropped quickly and was restored to the starting level when the buffer was switched back to the starting concentration. We then equilibrated the same cells with 1 mM glucose and treated them with 10 μM Glutor. As with the multi-well imager, we observed that fluorescence decreased and then rebounded (fig. S4B). Using the previous glucose exchange experiment as a crude calibration tool, we can approximate that the cytosolic concentration of glucose after Glutor treatment rebounds to a bit more than 10 μM.

The most obvious candidate for glucose storage is the ER, which has been previously interrogated by the FRET-based sensor FLIPglu in HepG2-immortalized liver cells (*11*). When iGlucoSnFR2 is expressed in the ER, the sensor appears to be saturated with 1 mM glucose in the external buffer, indicating that glucose is more concentrated in the ER than it is in the cytosol (Fig. 2C), in contrast to the prior observation that cytosolic and ER glucose concentrations in HepG2 cells are the same (*11*). After treatment with Glutor, the concentration of glucose in the ER drops, indicating that glucose could be exiting the ER (see the “Subcellular resolution of glucose transport between the cytosol and ER” section below), consistent with other GLUTs being present on the ER (*29*).

### Validation of iGlucoSnFR2 in cell culture—Glucose repletion and consumption rate

**Fig. 3.**
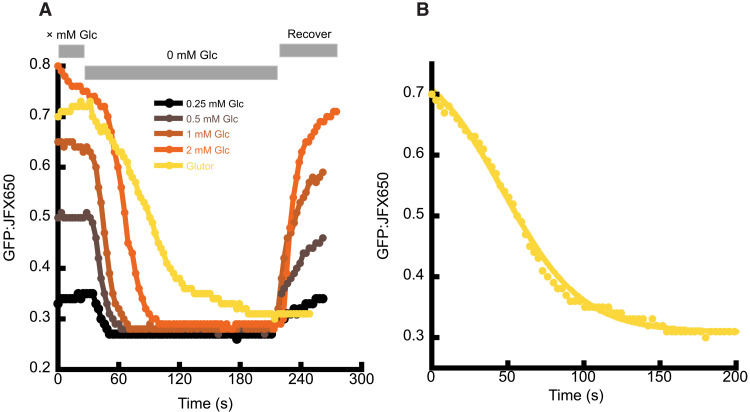
iGlucoSnFR2 reports changes in cytosolic glucose concentration with glucose removal or inhibition of glucose transport. (**A**) Observed changes in cytosolic glucose concentration as buffer is cycled from 0.25, 0.5, 1, or 2 mM glucose to buffer with 0 mM glucose or 2 mM glucose with addition of Glutor to 10 μM. (**B**) Data from the Glutor experiment at left replotted to place the time of Glutor addition to *t* = 0. Line represents a curve fit to the equation in the text. The best fit yields a *K*_d_ of 310 μM and a *k*_obs_ of 0.039. Data points are the average green/far-red ratio of the same 20 cells in the FOV.

The consumption rate of glucose is too rapid to be measured in the multi-well plate reader, but it can be observed in individual cells with constant perfusion of varying concentrations of glucose (Fig. 3A). We equilibrated HeLa cells expressing (cyto).iGlucoSnFR2.HaloTag-JFX650 with 0.25, 0.5, 1, or 2 mM glucose and then switched to buffer containing 0 mM glucose. With uninhibited bidirectional GLUT transporters, fluorescence dropped to baseline within a few seconds. When we equilibrated with 2 mM glucose and then added the GLUT inhibitor Glutor (10 μM), cells took approximately 2 min to drop to baseline fluorescence, indicating a consumption of 1 mM glucose/min, which agrees with measurements by enzyme-based bioanalysis instruments (recalculated from reported 0.2 pmol per cell per hour as ~1.3 mM/min) (*30*–*32*). However, the change in fluorescence of iGlucoSnFR2 is a function not only of the consumption of glucose but also the sensor’s ability to detect it. By substituting a single exponential decay function ([*L*] = *Ae*^−*kt*^) for [*L*] into the standard single site binding isotherm [*L*]/(*K*_d_ + [*L*]), we were able create a model that fits the observed change in fluorescence upon treatment with Glutor (Fig. 3B).

### Subcellular resolution of glucose transport between the cytosol and ER

**Fig. 4.**
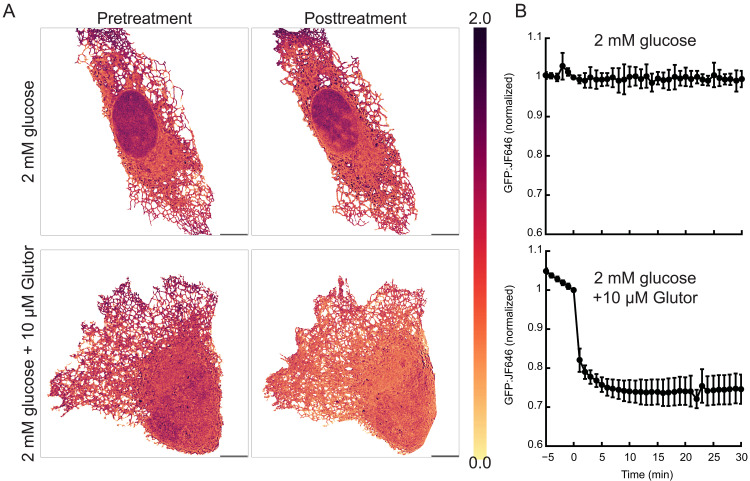
Response of ER-targeted iGlucoSnFR2-HaloTag to glucose levels in the ER. (**A**) Representative images of green to far-red fluorescence of U2OS cells transiently expressing (ER).iGlucoSnFR2-HaloTag-JF646 before and after treatment with 10 μM Glutor. Scale bars, 10 μm. (**B**) Mean and SD of normalized (ER).iGlucoSnFR2:JF646 responses (entirety of the ER) for untreated (top, *N* = 7) and treated (*N* = 6) cells. Treatment added at *t* = 0.

To confirm that glucose leaves the ER during blockade of plasma membrane glucose transport by Glutor, we imaged U2OS cells expressing (ER).iGlucoSnFR2.HaloTag-JF646 at 63× with Airyscan confocal imaging. (JF646 is more fluorogenic than JFX650, and better suited for high resolution ER imaging.) By making a heatmap of fluorescence (relative to JF646, to control for varying sensor concentration), we first observe that there is higher iGlucoSnFR2 fluorescence at the cell periphery and at the peri-nuclear ER than in the bulk of the ER (Fig. 4A) when cells are equilibrated in media containing 2 mM glucose. The addition of Glutor results in a decrease in fluorescence throughout the ER (Fig. 4, bottom panels). Because the mechanisms of glucose transport from the ER to the cytoplasm have not been completely elucidated, we anticipate that iGlucoSnFR2 will be useful for investigating the functional significance of heterogeneous glucose concentrations within the ER.

### Validation of iGlucoSnFR2 in cell culture—Gluconeogenesis in primary hepatocytes

**Fig. 5.**
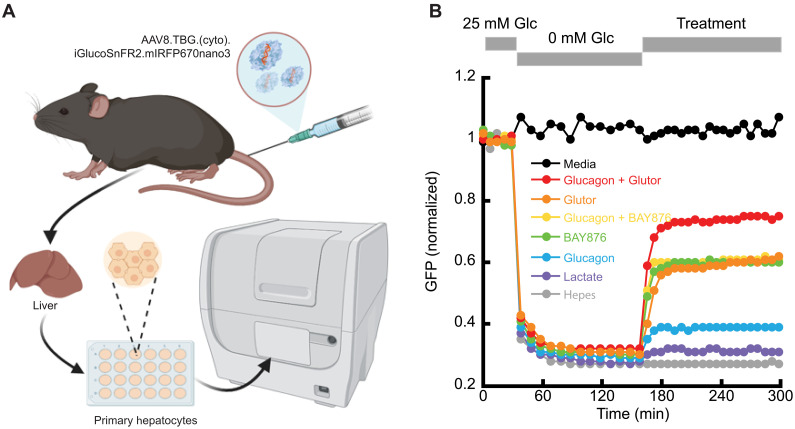
iGlucoSnFR2 reports gluconeogenesis in hepatocytes in response to stimulating treatments and inhibition of glucose transport. (**A**) Schematic of experimental set up to measure gluconeogenesis in primary hepatocytes. (**B**) Average normalized fluorescence of primary hepatocytes expressing (cyto).iGlucoSnFR2.mIRFP670nano3. Cells were imaged in primary culture media (with 25 mM glucose) for 30 min, and then the medium was replaced with Hepes buffer (0 mM glucose) for 2 hours. Gluconeogenic substrates (and glucose transport inhibitors) were added at the time indicated by the gray bar. Treatments were: No treatment (gray), 2 mM sodium pyruvate + 20 mM sodium lactate (purple), 2 mM sodium pyruvate + 100 nM glucagon (blue), 2 mM sodium pyruvate + 5 μM BAY876 (green), 2 mM sodium pyruvate + 100 nM glucagon +5 μM BAY876 (yellow), 2 mM sodium pyruvate + 5 μM Glutor (orange), and 2 mM sodium pyruvate + 100 nM glucagon + 5 μM Glutor (red). Cells maintained in glucose rich media for the entirety are in black. Data are represented as mean of 60 cells. SDs are removed for clarity but are presented in fig. S5. Unpaired *t* tests yield one-tailed *P* values of: Glucagon + Glutor versus Glutor, 0.002; glucagon + Glutor versus glucagon + BAY876, 1.3 × 10^−13^; glucagon + Glutor versus BAY876, 1.5 × 10^−12^; lactate versus Hepes, 0.02; glucagon versus Hepes, 3 × 10^−12^.

In addition to reporting cytosolic (and ER) glucose depletion through inhibition of glucose transport (and continued glucose consumption), we were able to observe gluconeogenesis in primary hepatocytes, the principal glucose regulating cells in the body. We infected mice with AAV8-expressing (cyto).iGlucoSnFR2.mIRFP670nano3 under control of the liver-specific thyroxine-binding globulin (TBG) promoter and harvested their livers and observed hepatocyte responses in a multi-well imager (Fig. 5). Cells were starved of glucose for 2 hours and then stimulated with pyruvate and lactate as gluconeogenic substrates or pyruvate and glucagon to stimulate gluconeogenesis. Both treatments increased iGlucoSnFR2 fluorescence; glucagon was more effective than pyruvate and lactate alone. Because the GLUTs facilitate bidirectional glucose transport both in and out of the cell, we queried whether blocking GLUTs (with BAY876, which blocks GLUT-1, or with Glutor, which blocks GLUT1, GLUT-2, and GLUT-3) would increase cytosolic glucose. Both drugs increased cytosolic glucose, with Glutor being more effective (Fig. 5, red and orange versus yellow and green). Glucagon increased glucose concentrations only with Glutor present (Fig. 5, red versus orange and yellow versus green).

### Cytosolic iGlucoSnFR2 outperforms iGlucoSnFR1 in vivo

**Fig 6.**
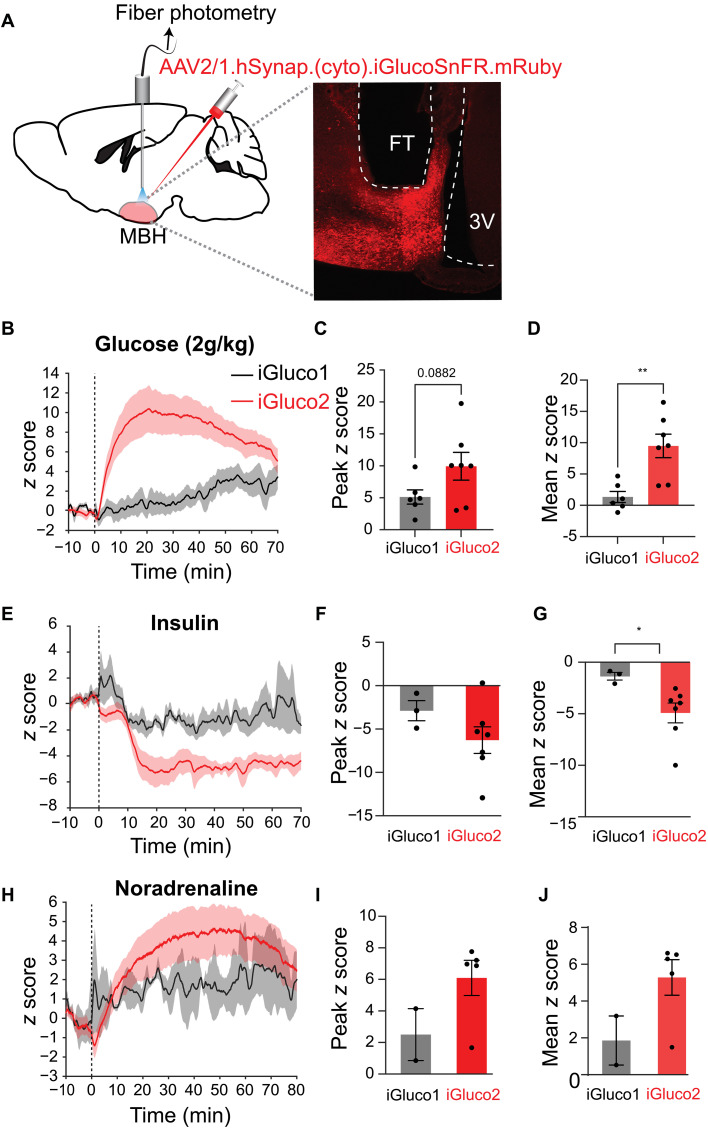
Comparing the sensitivity of detecting in vivo neuronal glucose dynamics between (cyto).iGlucoSnFR2 versus (cyto).iGlucoSnFR1. (**A**) Schematic of the fiber photometry recordings used for probing glucose dynamics in MBH neurons. A representative photomicrograph showing the fluorescent reporter expression iGlucoSnFR2 tagged with mRuby3 (red). (**B**) Change in iGlucoSnFR-dependent fluorescence signal in response to glucose administration (i.p., 2 g/kg) in overnight-fasted mice. (**C** and **D**) Quantification of the average peak and mean (15 to 30 min). (**E** to **G**) Same as in (B) and (D) except that insulin was injected (1 U/kg) after 3 hours of fasting during the light phase. (**H** to **J**) Same as in (B) to (D) except that norepinephrine was injected (2 mg/kg). Two-tailed, unpaired Student’s *t* tests. Error bars represent the SEM, *P < 0.05 and **P < 0.005. Unlabeled bar graphs, *P* > 0.05.

Previously, it was shown that iGlucoSnFR1 could report changes in intracellular glucose throughout *Drosophila* brain explants when the concentration of glucose in the surrounding buffer was modulated (*18*). It showed that in living zebrafish embryos, cytosolic glucose in muscle cells is well regulated, with small fluctuations that occur in concert with muscle activation, but that in starved fish, glucose homeostasis was lost. However, the utility of iGlucoSnFR1 in vivo in mammals was not demonstrated. With the improved response of iGlucoSnFR2 in cell culture models, we sought to compare cytosolically targeted iGlucoSnFR1 and iGlucoSnFR2 in the mediobasal hypothalamus (MBH) of mice (Fig. 6A), a brain region critical for controlling energy balance and glucose homeostasis. AAV-encoding (cyto).iGlucoSnFR2.mRuby3 or (cyto).iGlucoSnFR1.mRuby2 was injected in the MBH. After 2 to 4 weeks, fluorescence from the MBH was monitored using fiber photometry. iGlucoSnFR1 fluorescence responded to bolus treatment with intraperitoneal glucose (Fig. 6, B to D) or insulin (Fig. 6, E to G), but the magnitude of change was modest (mean of only 2 SD away from baseline) and noisy. In contrast, iGlucoSnFR2 exhibited clear and robust responses to treatments, with 5 to 10 SD changes in fluorescence (Fig. 6, B to G). Notably, iGlucoSnFR2 also reported changes in cytosolic glucose in the MBH upon treatment with noradrenaline, which is known to increase plasma glucose levels by stimulating hepatic glucose production, glucagon release, and suppression of insulin release (*33*). These findings establish that iGlucoSnFR2 is a more sensitive and reliable tool than iGlucoSnFR1 and can be used for observing cytosolic glucose dynamics in mammalian brain in vivo.

### iGlucoSnFR2 reports changes in glucose concentration in the extracellular space of the brain in response to chemical or electrical stimulation

One limitation of iGlucoSnFR1 is that it was difficult to effectively target it to the plasma membrane (facing the extracellular space) of mammalian cells. The modifications we have identified that improve translocation to the plasma membrane with other sensors (using SFGFP and codons optimized for expression in rodents) were beneficial also to iGlucoSnFR2. In cultured primary neurons, (mem).iGlucoSnFR1 failed to get to the membrane at all (fig. S1H), while (mem).iGlucoSnFR2 achieved greatly improved membrane localization (Fig. 1C). In neuronal cell culture, the membrane-localized sensor (facing the culture media) responds immediately to changes in extracellular glucose concentration (either up or down), while the cytosolic sensor responds slightly slower, as glucose transport out of the cell is slowed by its passage through GLUT transporters (fig. S6).

**Fig. 7.**
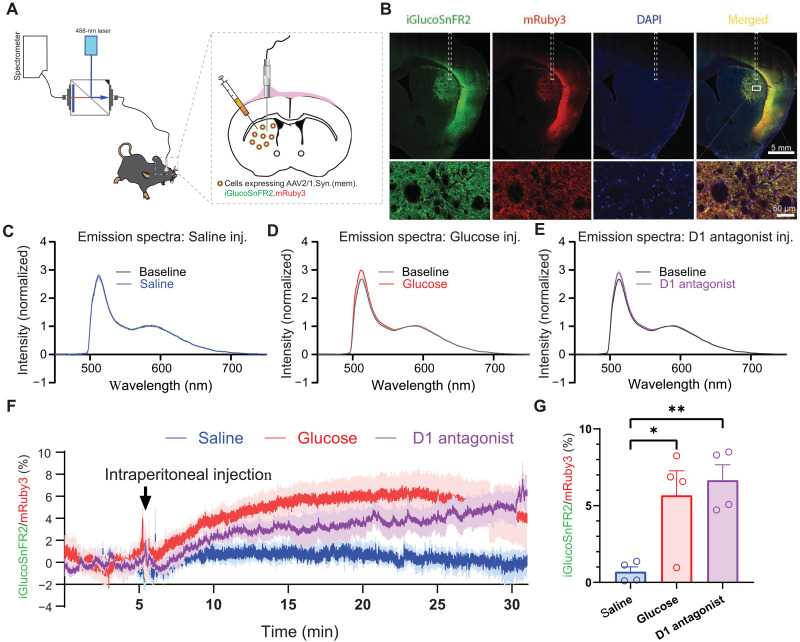
Striatal extracellular glucose dynamics measured in freely moving mice. (**A**) Schematic of the experimental setup. (mem).iGlucoSnFR2.mRuby3 was virally expressed in the dorsolateral striatum (DLS) and spectrally resolved fiber photometry was used to monitor fluorescence changes in freely moving mice. (**B**) Representative immunofluorescence images confirming expression of iGlucoSnFR2 (green, anti-GFP) and mRuby3 (red, anti-RFP) in the striatum. 4′,6-diamidino-2-phenylindole (DAPI) staining (blue) marks nuclei. High-magnification images of the ROI box in merged image (bottom row) show membrane-localized expression of the sensor. White dashed rectangle indicates the location of the fiber track. Scale bars, (top) 1 mm and (bottom) 50 μm. (**C** to **E**) Emission spectra of (mem).iGlucoSnFR2.mRuby3 normalized to mRuby3 showing fluorescence changes in response to saline (C), glucose (1 g/kg) (D), and D1 receptor (0.4 mg/kg) antagonist SCH23390 (E) injections. Traces show summarized spectra at baseline (gray) and postinjection (blue, red, and purple, respectively). (**F**) Time-lapsed photometry traces showing the percent changes of iGlucoSnFR2 to mRuby3 coefficients ratio in freely moving mice in response to systemic injections of saline (blue), glucose (red), or SCH23390 (purple). Black arrow above indicates the time of saline/glucose/SCH23390 administration. (**G**) Quantification of peak of the percent changes of iGlucoSnFR2 to mRuby3 coefficients ratio shows a significant increase in extracellular glucose levels following glucose and SCH23390 injection compared to saline (*P < 0.05 and **P < 0.01, one-way ANOVA). Data are means ± SEM; each dot represents an individual animal.

With this level of plasma membrane expression, we were able to assess whether iGlucoSnFR2 could be used to monitor extracellular glucose dynamics in freely moving mice. We expressed (mem).iGlucoSnFR2.mRuby3 in neurons of the dorsolateral striatum (Fig. 7A). Immunostaining confirmed the expression of (mem).iGlucoSnFR2.mRuby3, which was localized to the neuronal membrane (Fig. 7B). We measured fluorescence in vivo with a spectrally resolved fiber photometry system (*34*) that can record the entire emission spectrum of both fluorophores. This enables spectral unmixing analysis, effectively removing potential cross-talk between channels and artifacts such as hemoglobin absorption (*35*). In freely moving mice, the sensor exhibited robust fluorescence increases in response to intraperitoneal injection of glucose and no response to injection of saline (Fig. 7, C and D and F and G).

Glucose is the primary fuel source for neurons. To examine the correlation between extracellular glucose dynamics and neuronal activity, we measured changes in iGlucoSnFR2/mRuby3 fluorescent intensity in the dorsal striatum in response to intraperitoneal injection of the dopamine D1 receptor-specific antagonist SCH23390 (halobenzazepine). The inhibition of neuronal firing by SCH23390 (*36*) reduces ATP demand, leading to decrease glucose utilization by neurons. This leads to an accumulation of extracellular glucose, which was detected by iGlucoSnFR2 (Fig. 7, E to G).

**Fig. 8.**
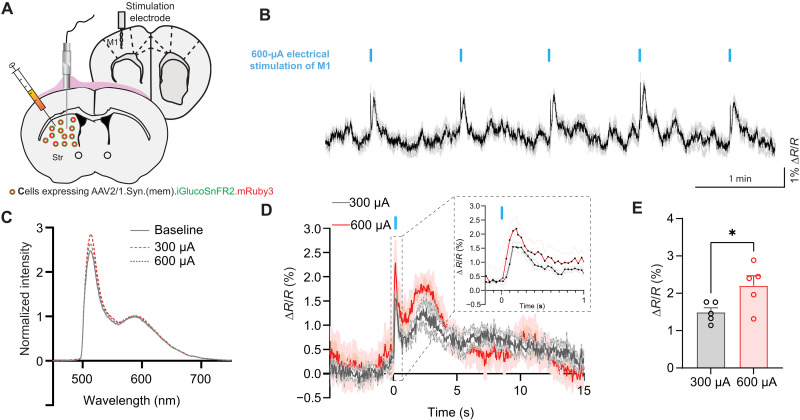
Effect of cortical stimulation on striatal extracellular glucose levels in freely moving mice. (**A**) Schematic illustration of the experimental setup. A stimulation electrode is implanted in the motor cortex (M1), and recording fiber is implanted in the striatum with (mem).iGlucoSnFR2.mRuby3 injection. (**B**) Average trace showing changes in extracellular glucose levels (∆*R*/*R*) over time during electrical stimulation of the motor cortex from five animals. Blue bars indicate the timing of electrical stimulation events. (**C**) Emission spectra of (mem).iGlucoSnFR2.mRuby3 at peak of stimulated fluorescence change, normalized to mRuby3 emission. (**D**) Average changes in extracellular glucose levels over time in response to electrical stimulation at two different current intensities (300 μA, gray; 600 μA, red). The blue bar indicates the timing of stimulation. Shaded areas represent the SEM. (**E**) Comparison of the peak iGlucoSnFR2 to mRuby3 coefficient ratios (∆*R*/*R*) between 300- and 600-μA stimulation intensities. Paired *t* tests were performed between groups. *P < 0.05. Data are presented as the means ± SEM.

While intracortical electrical stimulation has been shown to increase cerebral blood flow to the postsynaptic area (*37*, *38*), there is currently no direct evidence of a consistent rise in extracellular glucose levels following this stimulation, largely due to the lack of sufficiently sensitive glucose sensors. To address this gap, we used (mem).iGlucoSnFR2.mRuby3 to measure striatal extracellular glucose dynamics in response to electrical stimulations of the primary motor cortex (M1), which provides glutamatergic projections to the striatum (Fig. 8A). The stimulation of M1 resulted in a rapid (within ~100 ms), transient increase in extracellular glucose in the dorsolateral striatum, with the 600-μA stimulation eliciting a significantly larger and more sustained response compared to a 300-μA stimulation (Fig. 8, B to E). Notably, a secondary increase in glucose was consistently observed around 1 s after stimulation in freely moving animals. The observed fluorescence ratio increases from (mem).iGlucoSnFR2.mRuby3 reflect a stimulation-intensity–dependent accumulation of extracellular glucose, likely driven by neurovascular coupling mechanisms following transient neuronal activation. These results provide direct evidence that intracortical stimulation modulates glucose dynamics in downstream brain regions and further validates the utility of iGlucoSnFR2 as a sensitive tool for real-time, in vivo metabolic sensing.

### iGlucoSnFR2 fluorescence can provide a quantifiable spatial map of glucose concentrations in vivo

**Fig. 9.**
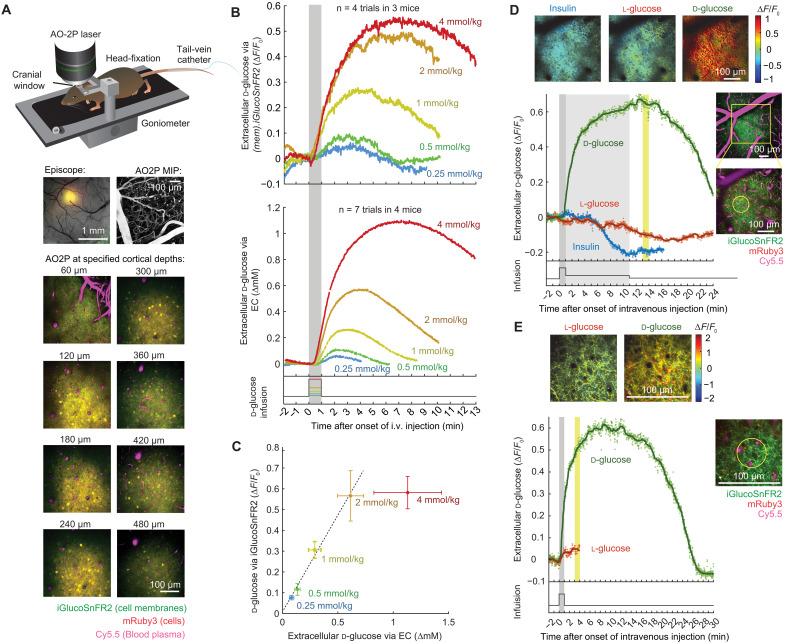
In vivo calibration of extracellular iGlucoSnFR2 fluorescence with electrochemically determined glucose concentration. (**A**) In vivo expression of AAV2/1.CAG.(mem).iGlucoSnFR2.mRuby3 after 16 days in neurons and astrocytes of sV1 cortex in awake mice. Top left: Fluorescence from iGlucoSnFR2 and mRuby3 overlaid on absorbance of green light. Top right: Maximum intensity projection (MIP) across 200 μm acquired by AO2P after retroorbital injection of Cy5.5. Bottom: AO2P three-dimensional stack at distinct cortical depths. (**B**) Sensitivity of iGlucoSnFR2 to detect changes in extracellular d-glucose was tested using increasing dosages of intravenous d-glucose in three awake mice at low blood glucose baseline [insulin, 2 IU/kg, i.v.). Mean of four trials; individual trials in fig. S7D. The same intravenous protocol in a separate set of four mice with EC probes. Mean of seven trials; for individual trials, see fig. S7E. (**C**) Peak signal increases were correlated with optical recordings from (B). (means ± SD, linear fit: 0.929*x + 0.005). (**D**) Injection of d-glucose (8 mmol/kg) via acute tail vein catheter led to 0.642 ± 0.017 SD Δ*F*/*F*_0_ increase (means ± SD for 3-min after injection relative to baseline 1 min before injection, yellow shade) of iGlucoSnFR2 in layer I of sV1 cortex. l-glucose injection led to a −0.072 ± 0.010 SD Δ*F*/*F*_0_ decrease of iGlucoSnFR2 signal. The injections were preceded by insulin (1 IU per kg bodyweight in 1 min, followed by 1 IU/kg in 15 min, −0.208 ± 0.009 SD). Top: Δ*F*/*F*_0_ for 3-min (yellow shade) postinjection (gray shade) is mapped for each pixel and injection. (**E**) Temporal transient (for yellow ROI) and spatial distribution of iGlucoSnF2 signal following d- and l-glucose (4 mmol/kg) in 1 min in a sV1 cortex region with three perpendicular capillaries (Cy5.5) in an awake mouse.

The experiments above show that iGlucoSnFR2 can report changes in intracellular or extracellular glucose deep in the brain using fiber photometry. To demonstrate that iGlucoSnFR2 can also be used for high-resolution imaging of glucose dynamics in vivo, we expressed it on the surface of both astrocytes and neurons (with the CAG promoter) in the barrel cortex and imaged up to 480-μm deep with two-photon fluorescence and adaptive optics (AO2P) to correct for aberration (Fig. 9A). Intravenous injection of d-glucose resulted in an immediate rise in the iGlucoSnFR2:mRuby3 fluorescence as expected, while insulin lowered it, and l-glucose showed no response (Fig. 9D). Labeling the vasculature with 2-megadalton–dextran conjugated dye Cy5.5 allowed us to avoid regions with increased hemoglobin absorption artifacts. Red mRuby3 emission was used for translational motion correction, which was not affected by enhanced bleaching of mRuby3 as compared to iGlucoSnFR2 (fig. S7C).

To calibrate membrane tagged iGlucoSnFR2 fluorescence in vivo, mice were fasted overnight, and insulin (2 IU/kg bodyweight) was injected intravenously via a catheter to achieve reproducibly low blood and extracellular glucose levels. Glucose was injected using syringe pumps in increasing dosages (0.25, 0.5, 1, 2, or 4 mmol/kg bodyweight), and the mean fluorescence increase was measured in mouse primary somatosensory cortex (sV1) (Fig. 9B). For in vivo calibration, the same infusion protocol was carried out in mice with an implanted electrochemical (EC) probe sampling extracellular space in sV1. EC probes were calibrated ex vivo at 37°C, allowing to measure absolute changes of d-glucose, resulting in a linear correlation of iGlucoSnFR2 with extracellular glucose concentrations between 0.08 and 0.62 mM (slope of 0.929) (Fig. 9C).

AO2P imaging of layer I sV1 cortex allowed us to spatially map changes in extracellular glucose upon intravenous injections in the awake mouse with 1-Hz temporal and submicrometer spatial resolution (Fig. 9D). By weighting each pixel brightness with the fluorescence intensity at a 1-min readout window, the average signal increase (∆*F*/*F*_0_) per pixel relative to baseline was mapped for insulin (2 IU/kg), l-glucose (8 mmol/kg) and d-glucose (8 mmol/kg) injection (Fig. 9D, top). The temporal dynamics were plotted for a region of interest void of overlaying vasculature as identified by vascular Cy5.5-dextran (Fig. 9D, bottom). Even higher spatial resolution was achieved for a zoomed-in recording in layer I sV1 with multiple small blood vessels perpendicular to the imaging plane (Fig. 9E). At both spatial scales, intravenously injected glucose distributed homogenously in the extracellular space, while iGlucoSnFR2 peaked minutes after blood levels (fig. S7F), indicating that d-glucose uptake in the brain is limited by transport rates across the blood-brain barrier rather than diffusion in the extracellular space.

## DISCUSSION

We have developed a second generation genetically encoded intensity-based glucose sensing fluorescent reporter (iGlucoSnFR2) that outperforms the original glucose sensor (iGlucoSnFR). We have validated its function in mammalian cell culture (including the endomembrane system) and in vivo, with perturbations that are known to affect glucose consumption or production resulting in the expected fluorescence changes. iGlucoSnFR2 has been targeted to the plasma membrane, cytosol, and ER, and we have demonstrated that it functions in vivo in mouse brain not only to gross perturbations such as bolus addition of glucose or insulin but also to upstream perturbations such as administration of noradrenaline or electrical stimulation. Last, we have shown that by pairing the fluorescence observations with EC glucose probes, we can construct a calibration curve to quantify glucose concentrations in the extracellular space.

The extraordinary importance of glucose as an energy source for the brain (it consumes 20 to 25% of person’s energy) and its homeostasis as maintained by the liver has been known for decades (*39*). Positron emission tomography (PET) imaging using [^18^F]fluorodeoxyglucose (FDG) has been able to map glucose metabolism at rest and during various tasks, with resolution at the millimeter scale and temporal resolution on the order of tens of seconds (*40*). While PET imaging is noninvasive and applicable to human studies, it requires large, expensive machinery and compliance from the subject to keep the head still. In vivo fluorescence imaging, on the other hand, is applicable to animal models, but with far higher resolution (both spatially and temporally) and at far lower instrumentation cost. Furthermore, having the fluorescent sensor genetically encoded means that specific subpopulations of cells (through the use of Cre dependent recombinases) can be imaged in isolation along with subcellular compartment localization, which can inform compartment models of glucose uptake allowing for more specific interpretation of dynamic FDG-PET data.

We anticipate that the results presented here will facilitate physiology experiments that until now have been impractical. For example, it is hypothesized that in some neurodegenerative diseases, neurons fail due to insufficient cellular energy (*4*). The development of pharmacological treatments that mobilize glucose metabolism to those specific neurons could be advanced with the availability of a glucose sensor capable of detecting changes in glucose concentrations at the cellular level in vivo. In addition to studying disease states, iGlucoSnFR2 offers opportunities to study the function of various cells in the brain as they function normally: How glucose is transported from the blood vessels to the neurons and glia, and how those cell types respond to changes in glucose.

For example, some theories of neurovascular coupling purport that local increases in blood flow and glucose delivery are tightly linked to neuronal activity (*41*). However, technical limitations—such as the low temporal resolution of microdialysis—have historically hindered real-time measurement of these rapid changes. Membrane-localized iGlucoSnFR2 enables subsecond, high-resolution monitoring of extracellular glucose dynamics. Using this tool, we observed an initial glucose increase within ~200 ms of electrical stimulation, followed by a slower rise peaking at around 3 to 4 s (Fig. 8D). This biphasic response reflects the two-phase model of neurovascular coupling: an early feed-forward phase and a later feedback phase dependent on metabolic byproducts. The slower glucose component aligns with the well-established astrocyte-mediated pathway, where glutamate activates Ca^2+^ signaling in astrocytes, leading to release of prostaglandins and nitric oxide (*42*, *43*).

More recently, a direct axo-vascular mechanism was reported (*44*) in which glutamatergic axons form synaptic-like contacts with arteriolar smooth muscle cells, allowing neurons to rapidly modulate vessel tone without glial intermediates. This fast, localized signaling likely underlies the early glucose rise we observed, which closely follows the electrostimulation-evoked glutamate synaptic activation that occurs within milliseconds to a few hundred milliseconds of electrical stimulation (*45*). Together, these results suggest that (mem).iGlucoSnFR2-mRuby3 detects the integrated effects of both direct neuronal signaling to vasculature and indirect astrocyte-mediated pathways, both of which can shape extracellular glucose availability during neural activity.

While we believe iGlucoSnFR2 will be used in many cellular physiology experiments, we are aware that are a few intrinsic limitations to intensity-based fluorescent sensors and aspects of the sensor that could be further improved. One of the benefits of ratiometric sensors, such as those based on FRET or fluorescence lifetime, is that if they can be calibrated, then enabling the concentration of the analyte of interest can be determined, regardless of the intrinsic brightness or expression level of the sensor. With an intensity-based sensor, factors that affect baseline brightness (expression level of the sensor and background amounts of the analyte) prevent direct correlation of concentration with intensity. For some of our original iSnFRs—such as iGluSnFR, iGABASnFR, and iAChSnFR—the determination of concentration was less important than the ability to see those neurotransmitters being released in an almost binary-like fashion, and thus ratiometry was not prioritized as a feature. However, with metabolic sensors, subtle changes in analyte concentration are more important. Ratiometric measurement with all the cpGFP-based sensors is possible due to an isosbestic point ~435 nm, as the excitation peaks of 405 and 485 nm shift between the bound and unbound state. However, excitation with 405-nm light is generally more damaging to tissue and does not penetrate as deeply as longer wavelength light. To address this limitation, we have made variants of iGlucoSnFR2 with C-terminal tags that are orange/red (mRuby3), far red (mIRFP670nano3), or amenable to synthetic fluorophores (HaloTag).

Another aspect of metabolic detection that will interest researchers as imaging techniques improve will be the ability to observe changes in multiple analytes in the same cells at the same time. FRET sensors occupy large spectral bandwidth and can generally only be used to measure one analyte at a time. While iGlucoSnFR2 is based on GFP, we anticipate that we will eventually be able to develop similar sensors using blue or red fluorophores for multiplex imaging.

## MATERIALS AND METHODS

### Mutagenesis, candidate screening, and subcloning

The DNA sequence encoding the glucose-binding protein of iGlucoSnFR1 was optimized for expression in mouse using the codon optimization tool of Integrated DNA Technologies (IDT). The cpeGFP sequence was replaced with that encoding cpSFGFP [copied from SF-iGluSnFR; (*46*)]. The linkers of iGlucoSnFR1 (linker 1 = +PA, linker 2 = +NP) were retained. The entire gene was obtained as a synthetic gBlock from IDT and cloned into a derivative of the pRSET bacterial expression vector (Invitrogen) via Bgl II and Pst I sites. Primers encoding libraries of the residues at the junction of the binding protein and the cpSFGFP were used to make libraries of variants using the uracil template method (*47*) and transformed into T7 Express cells (New England Biolabs). Individual colonies were picked into single wells of a 96-well plate (2.2 ml; Arctic White Square Well Polypropylene, AWLS-T110-10) containing 850 μl of autoinduction media (*48*) and ampicillin (100 μg/ml) and set to shake for 18 hours at 30°C at 350 rpm. Bacterial pellets were collected by centrifuging the 96-well plates at 3000 rpm in a swinging bucket rotor. Supernatant was removed, replaced with 500 μl of phosphate-buffered saline (PBS), and vortexed to resuspend the bacterial pellets. The collection and resuspension process was performed three times to remove endogenous glucose. Pellets were frozen at −20°C overnight. Clarified lysate was obtained by thawing the pellets with 1 ml of PBS, resuspending by vortex, and subsequent centrifugation. A 100 μl of lysate was transferred to black flat bottom 96-well plates by multichannel pipet. GFP fluorescence was measured in a Tecan Safire 2 fluorescence plate reader [excitation of 485 nm (20-nm bandpass), emission of 535 nm (20-nm bandpass)], and glucose was added to 10 mM and then measured again. Candidates with higher than starting point ∆*F*/*F* were isolated, sequenced, and carried forward for additional mutation and screening. The variant with the highest ∆*F*/*F* was declared to be iGlucoSnFR2. C-terminal tags encoding HaloTag, mIRFP670nano3, and mRuby3 were subcloned into the bacterial expression vector via the Pst I site. AAV plasmids were constructed ad hoc to provide the promoter, subcellular targeting, and normalization tags as needed.

### Protein purification and in vitro characterization

Methods relating to Fig. 1 and fig. S1. Bacterial expression plasmids encoding the iGlucoSnFR2.C-TerminalTag proteins were transformed into T7 express cells. Individual colonies were used to inoculate 300 ml of autoinduction media with ampicillin (100 μg/ml) in a 2-liter flask and grown at 30°C for 18 hours at 225 rpm. Cells were pelleted by centrifugation at 2000*g*, resuspended in 30 ml of PBS, and frozen at −20°C overnight. Bacterial suspension was thawed in room temperature water, sonicated for 5 min (5-s on, 5-s off) on ice. The cellular debris was removed by centrifugation at 6000*g* for 10 min. The supernatant was transferred to a clean tube and clarified by centrifugation at 35,000*g* for 60 min. Clarified lysate was loaded onto a 5-ml IMAC Fast Flow column (Cytiva) at 2 ml/min, rinsed with 40 ml of PBS, and eluted with a gradient to 150 mM imidazole over 60 min. Fluorescent green fractions were pooled, concentrated by centrifugal ultrafiltration, and dialyzed with PBS to remove any residual glucose. Protein concentration was determined by diluting 2 μl of protein into 18 μl of 1 M NaOH, measuring absorbance at 447 nm, and using the extinction coefficient of GFP = 44,000 μM^−1^ cm^−1^.

All in vitro characterization was performed with 0.2 μM protein in PBS at room temperature. Glucose titrations were performed in a Tecan Safire 2 plate reader [excitation of 485 nm (20-nm bandpass), emission of 535 nm (20-nm bandpass)] by making serial dilutions of a concentration stock of 1 M glucose and adding them to 100 μl of purified protein. Excitation spectra were collected by observing emission at 515 nm (5-nm bandpass) while varying excitation wavelength (5-nm bandpass) from 300 to 500 nm. Emission spectra were collected by exciting at 485 nm and observing emission (5-nm bandpass) from 500 to 600 nm. Kinetic measurements were made by mixing equal volumes protein with glucose in a SX-20 stopped flow fluorimeter (Applied Photophysics) five times and averaging the traces.

### Inhibition of glucose transport in HeLa cell culture

Methods relating to Figs. 2 and 3. Cell culture maintenance and electroporation were performed by the Howard Hughes Medical Institute at the Janelia Research Campus, Immortalized Cell Line Culture Shared Resource Core Facility (RRID:SCR_026515). HeLa cells [American Type Culture Collection (ATCC), CCL-2] were cultured in Dulbecco’s modified Eagle’s medium (DMEM) supplemented with 10% fetal bovine serum (FBS), 1% l-glutamine, and 1% penicillin/streptomycin and incubated in a CO_2_ incubator at 37°C with 5% CO_2_.

For transfection, the reaction mixture was prepared by combining 1 × 10^6^ HeLa cells with 0.5 μg of each plasmid per reaction in 20 μl of electroporation SE solution (Lonza, V4SC-1096). Each reaction was transferred to a 96-well electroporation plate, and electroporation was performed according to the manufacturer’s protocol. Subsequently, 2.5 × 10^5^ cells per well were plated in a 24-well plate and further incubated until imaging.

Approximately 18 hours after transfection, JFX650-HaloTag ligand was added to the growth media from a 1 mM stock to a final concentration of 0.1 μM. One hour later, medium was removed, and cells were washed twice with Hepes buffer [20 m Hepes, 119 mM NaCl, 2 mM CaCl_2_, 2 mM MgCl_2_, and 2.5 mM KCl (pH 7.5)] and equilibrated with Hepes buffer plus glucose (per experimental conditions); buffer was changed or added to at the set time points. Cells were imaged in a Cytation 5 multi-well cell imager (Agilent Biotek) with a 10× objective and excitation with 469 nm (35-nm bandpass) and emission of 525 nm (39-nm bandpass) for GFP. For Imaging JFX650, a Cy5 filter cube with excitation of 628 nm (40-nm bandpass) and emission of 685 nm (40-nm bandpass). We imported stacks of Cy5 images into Fiji, made into a Z-axis average projection, and then threshold was adjusted to the point where the Cy5 mask appeared to mostly overlap with the GFP fluorescence and then dilated by 3 pixels twice. Regions of interest (ROIs) were defined by analyze particles using 400 to 8000 pixel cutoff values, and the mean fluorescence per ROI per image was exported to a spreadsheet. Corresponding GFP images were imported, and their mean fluorescence was determined using the same ROIs and exported to a spreadsheet. Ratios of GFP to Cy5 fluorescence were calculated per ROI and averaged.

### U2OS cell ER scale glucose dynamics

Methods relating to Fig. 4. The U2OS is a human osteosarcoma cell line, obtained directly from ATCC (HTB96). The cells were maintained in complete phenol red-free DMEM (Corning) supplemented with 10% (v/v) FBS (Corning), 2 mM l-glutamine (Corning), penicillin (100 IU/ml), and streptomycin (100 μg/ml; Thermo Fisher Scientific) at 37°C in 5% CO_2_ and passed with 0.25% (w/v) Trypsin EDTA (Corning). Glass-bottom dishes were precoated with fibronectin. Cells were incubated with 200 nM JF646-HaloTag ligand for 30 min.

Live cell imaging was carried out in phenol red-free DMEM using Zeiss 980 LSM with Airyscan, plan-apochromatic 63× oil objective [numerical aperture (NA) = 1.4]. Cells were illuminated with 488 and 639 nm at <2% laser power using Line Scan modality at a framerate of 5 min. Images were preprocessed with Airyscan processing ZEN software (Zeiss). Cell and ER segmentation and ratiometric analysis were done using a custom code available on github (github.com/monikamakurath/pythonRatiometricAnalysis). Image acquisition started in complete glucose media, and glucose, glucose-free, or glucose-free with 10 μM glutor media was introduced via manual perfusion between frames 8 and 12. The glucose-free medium was balanced for osmolarity changes with 25 mM mannitol. The mean ratio intensity time series was shifted such that first frame after media exchange is at *t* = 0 and ratio intensity = 1.

### Gluconeogenesis in primary hepatocytes

Methods relating to Fig. 5. Primary hepatocytes expressing iGlucoSnFR2.mIRFP were isolated from 8-week-old C57BL/6J male mice using a two-step collagenase perfusion method. Briefly, AAV containing the construct under a liver-specific TBG promoter was injected into the animals via the tail vein at 2 × 10^11^ GC (genome copies) per animal. After 48 hours, the mice were anesthetized with isoflurane, and the liver was perfused through the inferior vena cava with perfusion buffer (Hanks’ balanced salt solution without calcium, magnesium, and phenol red; add 0.5 mM EDTA and 25 mM Hepes), followed by digestion with collagenase (25 μg/ml; Liberase). The liver was subsequently harvested, dissociated in cold plating media (William’s E-medium with 2 mM GlutaMAX, 1× penicillin/streptomycin, 2 mM sodium pyruvate, 0.1 μM insulin, 1 μM dexamethasone, and 10% FBS), and filtered through a 70-μm strainer. Hepatocytes were pelleted by centrifugation at 50*g* for 2 min, followed by 45% Percoll gradient centrifugation at 200*g* for 10 min. Viable cells were then plated in collagen-coated glass-bottom 24-well plates (10 μg/cm^2^) using plating media and incubated under humidified conditions at 37°C with 5% CO_2_. Plating medium was replaced with maintenance media (same as plating media, but with 0.1 nM insulin and 0.1 μM dexamethasone) after 3 hours. Experiments were conducted after 24 hours of incubation.

Isolated primary hepatocytes were imaged using a Cytation 5 cell imager (Agilent Biotek) with a 20× objective (LUCPLFLN, Olympus, NA = 0.45). Fluorescence was excited using a lamp source with an excitation filter (469 nm (35-nm bandpass), and emission was collected through a 525 nm (39-nm bandpass) emission filter using a photomultiplier tube detector. Baseline fluorescence was acquired from a set field of view, and then the cells were washed twice with Hepes buffer [20 mM Hepes, 119 mM NaCl, 2 mM CaCl_2_, 2 mM MgCl_2_, and 2.5 mM KCl (pH 7.2)] and incubated in Hepes buffer for 2 hours without glucose. During the starvation period, the cells were imaged every 10 min. Cells were then treated with gluconeogenic substrates as listed in the figure legend in the presence of 2 mM sodium pyruvate. Fluorescence images were acquired for each condition, and the integrated density from single cells were quantified using FIJI software. Fluorescence counts were normalized with respect to baseline fluorescence for all the conditions.

### In vivo comparison of (cyto).iGlucoSnFR1 and (cyto).iGlucoSnFR2 in MBH

Methods relating to Fig. 6.

#### Stereotaxic injections

Stereotaxic surgeries were performed on adult mice (5 to 6 months old) as described previously (*49*). Briefly, mice were anesthetized with 1.5% isoflurane and secured in a stereotaxic instrument (David Kopf Instruments, Tujunga, CA). After disinfecting the scalp, a small incision was made to expose the skull, and a cranial burr hole was drilled at the designated injection site. Using a pulled glass pipette (Drummond Scientific, Wiretrol, Broomall, PA) with a tip diameter of ~50 μm, 150 to 200 nl of virus were injected into the MBH at coordinates relative to bregma: anteroposterior (AP), −1.2 mm; mediolateral (ML), ±0.37 mm; and dorsoventral (DV), −5.8 mm. Injections were carried out at a controlled rate of 50 nl/min using a micromanipulator (Narishige, East Meadow, NY), with a 10-min diffusion period following each injection. After the injection, the glass pipette was carefully withdrawn, an optical fiber was implanted at the injection site and was secured with acrylic cement. Mice were allowed a recovery period of 2 to 4 weeks to ensure proper healing and transgene expression before commencing experiments.

#### Fiber photometry

Freely behaving mice were connected to fiber optic cable (400-μm core diameter, 0.48 NA, bundled fibers; Doric Lenses) via ferrule implants using ceramic mating sleeves (Thorlabs), which were covered with black tubing to minimize light interference. Mice were allowed to acclimate to the tethering system for 1 day before fiber photometry experiments and were tethered for at least 30 min before recordings. During this acclimation period, mice received saline intraperitoneally to prepare them for subsequent intraperitoneal injections. Fiber photometry signals were recorded at a sampling rate of 3 Hz using the Doric FP Bundle Imager (Doric Lenses). Light intensity at the fiber tip was adjusted to ~30 to 50 μW for each wavelength. Baseline recordings lasted 10 to 30 min before intraperitoneal injections, followed by at least 30 min of postinjection recording. At the conclusion of the experiments, mice underwent post hoc histological evaluation to confirm the accuracy of viral transgene expression and fiber placement. Animals with off-target expression or misplaced fiber tips were excluded from the analysis.

For the analysis, the signal obtained by 405-nm wavelength was linearly fit to the signal obtained by 465-nm wavelength using a custom MATLAB script based on a linear least squares approach. The Δ*F*/*F* values were then calculated as to normalize differences in signal intensities across animals, *z* scores were computed using the formula [*F* − μ(baseline)]/δ(baseline), where *F* is the given signal at time and μ and δ are the mean and SD of the baseline period, respectively.

### In vivo spectrally resolved fiber photometry of (mem).iGlucoSnFR2

Methods relating to Figs. 7 and 8.

#### Mice

All animal protocols were approved by the US National Institute of Environmental Health Sciences Animal Care and Use Committee. Experiments were carried out using 2- to 4-month-old male and female mice. C57BL/6J (#000664) mice were obtained from the Jackson Laboratory. All mice housed under reverse light cycle conditions and had access to water and food ad libitum. Mice were housed two to four per cage before optical probe implantation surgery and singly housed after surgery.

#### Stereotaxic injection

AAV2/1.hSynap.(mem).iGluocoSnFR2.mRuby3 was microinjected into the left or right dorsal lateral striatum by standard stereotaxic procedures with animals under isoflurane anesthesia (*34*). The coordinates used for targeting the dorsolateral striatum (DLS) were AP, +0.50 mm; ML, ±2.30 mm from Bregma; and DV, −2.75 mm from the brain surface. A total volume of 1.0 μl of AAV vectors per site was injected at the rate of 0.1 μl/min through a Hamilton Neuros syringe with a 30-gauge needle. The needle was left in place for five more minutes before withdrawal.

#### Optical fiber probe and microelectrode implantation

One month after the AAV injection, mice underwent the second stereotaxic surgery to receive fiber probe implantation. One or two bur holes were drilled through the skull to target the dorsal striatum unilaterally or bilaterally (AP, +0.50 mm; ML, ±2.40 mm from bregma) using a #1/2 (0.027″ diameter) drill bit. Another pair of bur holes for anchoring screws were drilled bilaterally above the parietal lobes. After the anchoring screws were in place, the homemade 105-μm core optical fiber probe was slowly lowered onto the cortical surface through the burr hole and then further lowered toward the dorsal lateral striatum at ~200 μm per step until the spectrum was detected. The probe was then lowered at 50 μm per step until the fluorescence intensity reached a plateau. The final tip location was ~2.2 mm below the brain surface. The probe was then fixed in place with a generous amount of dental acrylic (Jet, Lang Dental Mfg. Co. Inc.). For simultaneous motor cortex (M1) electoral stimulation and DLS fiber photometry, a twisted bipolar stainless steel electrode (MS303/3-B/SPC, Plastics One, USA) was implanted at a 15° angle in M1 region using the following coordinates: AP, +1.20 mm; ML, ±1.50 mm; DV, −1.0 mm from brain surface, in the same hemispheres as the DLS fiber implant. The animals were allowed to recover for 1 week before experiments proceeded.

#### Spectrally resolved fiber photometry

Emitted photons from fluorescent sensors were collected by a spectrometer as described previously (*34*). The in vivo recordings in awake behaving mice were carried out in an open-top mouse operant chamber (21.6 cm by 17.8 cm by 12.7 cm, Med Associates) housed in a sound attenuating box. Fluorescence spectra were acquired using 19-ms integration time and were triggered by 25-Hz TTL pulses sent from a digital output module (DIG-726TTL, Med Associates) on a customized mouse operant conditioning package from Med Associates. The output power of the 473-nm laser measured at the end of patch cable was set at about 50 to 75 μW. Spectral linear unmixing was carried out using a customized program written in R.

#### Intracortical electrode stimulation in freely moving mice

For evoke cortico-striatal postsynaptic potentials in dorsolateral neurons, a train of stimulating pulses (5 pulses at 50 Hz with 200-μs pulse duration) at 300 or 600 μA were delivered every 1 min using a constant isolated stimulator (DS3, Digitimer, Ltd) controlled by a waveform generator (SDG1025, SIGLENT TECHNOLOGIES). The timing of stimuli was controlled by TTL input from Med Associates.

#### Immunohistochemistry

Mice were given a lethal dose of sodium pentobarbital and transcardially perfused with cold PBS followed by 4% paraformaldehyde (PFA). Brains were post-fixed in 4% PFA for 24 hours and then transferred to 30% sucrose in PBS for storage at 4°C until further processing. Frozen coronal slices were sectioned on a microtome with a freezing unit (KS34, Thermo Fisher Scientific) at the thickness of 35 μm. Sections were blocked with 10% normal goat serum (VectorLabs) and permeabilized with 0.1% Triton X-100 (Sigma-Aldrich) for 1 hour at room temperature, followed by incubation at 4^o^ C overnight with primary antibodies against GFP (1:1000; ab13970, Abcam) for detecting iGlucoSnFR2 and anti–red fluorescent protein (RFP) (1:1000; ab62341, Abcam) for detecting mRuby3. After washing out excessive primary antibodies with PBS, the slices were incubated in the secondary antibodies for 2 hours at room temperature. The secondary antibodies used were Alexa Fluor 488–conjugated goat anti-chicken (1:500; A-11039, Invitrogen) and Alexa Fluor 568–conjugated goat anti-rabbit (1:500; A-11036, Invitrogen). After washing, the slices were mounted on slides and imaged on a Zeiss Axiocam MR monochrome camera installed on Axio observer Z1 fluorescent microscope with 20× objective (NA = 0.8). The images were acquired and processed using Zen 2012 Blue software (Carl Zeiss).

### In vivo imaging of (mem).iGlucoSnFR2 by two-photon fluorescence microscopy

Methods relating to Fig. 9.

#### In vivo imaging

Awake imaging was done in male and female wild-type mice (bred from C57BL/6J from the Jackson Laboratory) at an age of 2 to 12 months. Mice were anesthetized with isoflurane using a precision vaporizer, 4% (v/v) in oxygen for induction, and 1 to 2% (v/v) for maintenance and given the analgesic buprenorphine subcutaneously (0.1 μg/g body weight). The animal was mounted on a stereotaxic frame, and the body temperature was maintained at 36° to 37°C with a heating pad during anesthesia. A 3.5-mm craniotomy was created over the right vS1 cortex (centroid at 1.5-mm posterior to the bregma and 3.4-mm lateral from the midline) while avoiding damage to the dura. Glass pipettes (Drummond, #QF100-60-10) were pulled (Sutter Instruments, #P-2000) and beveled to a tip at 10- to 20-μm outer diameter with fine scissors. Within primary somatosensory barrel cortex (vS1) cortex, 50 to 150 nl of AAV (AAV2/1.hSynap.CAG.(mem).iGlucoSnFR2.mRuby3 at a titer of 10^12^ GC ml^−1^) were injected 450 μm below the dura using a motorized manipulator (Sutter Instruments, #MPC-200, #MP285/M-21826, and #ROE-200) using a syringe pump (KD Scientific, #Legato 185). A cranial window was custom-made with a 3.5-mm round coverslip (no. 1 CS-3.5R, Warner Instruments, #64-0739) and was glued to a 5-mm round coverslip (no. 1 CS-5R, Warner Instruments, #64-0700) using Norland Optical Adhesive NOA 61, embedded in the craniotomy, and sealed with cyanoacrylate glue (Loctite, #401). Metabond (Parkell L-Powder and Quick Base) was further applied around the edge to reinforce stability. A custom-designed titanium head bar was attached to the scalpel-scratched skull with Metabond, and the remaining exposed bone was covered with dental acrylic (Lang Ortho-Jet).

In vivo imaging was carried out after 1 to 6 months of expression and at least three 1-hour sessions of habituation for head fixation. Prolonged AAV expression led in some cases to the formation of fluorescent crystal sheet, which were excluded. All imaging experiments used head-fixed awake mice under AO2P with correction using a deformable mirror for aberration originating in the microscope73. The femtosecond-pulsed laser Chameleon Discovery NX (Coherent) was tuned to 800 to 1050 nm for excitation. A 25× objective (Olympus, XLPlanN); dichroic mirrors (FF775-Di01 and FF662-Di01); specific filters for green (505AF50 and E680), red (FF01-593/46 and E680), and far-red (FF01-708/75 and FF01-770/SP); and MPPC modules (Hamamatsu, 20A-002) were used for emission detection at 1 Hz on a resonant-galvo scanner. Post-objective power was kept under 40 mW for all measurements. The dura was aligned to the imaging plane using two goniometers (GNL10/M) under an episcope (Zeiss Stereo, X-Cite 120Q). Tail vein catheters (custom-made 30-gauge needle connected to PE10 tubing) were placed under isoflurane 1.5% and after 5 min of tail warming at 37°C, and successful intravenous placement was tested using 50 μl of saline injection before letting the mouse wake up. Calibrated syringe pumps were connected using PE10 tubing and Y-connectors (Instechlab, #SCY-25) for d-glucose (Sigma-Aldrich, G8270) and l-glucose (Sigma-Aldrich, G5500) infusions.

For in vivo calibration, a guide cannula was implanted above the vS1 cortex in four mice. After a week, an EC probe specific to d-glucose (Pinnacle Technology, #7004-80) was precalibrated ex vivo using d-glucose and ascorbic acid at 37°C and was inserted into the cortex under isoflurane 2%. After 4 hours of recovery, a tail vein catheter was placed under isoflurane 2% for awake imaging for insulin and repeated d-glucose 20% (w/v) injections. Mice were fasted for 18 hours overnight before imaging. Venous blood was sampled from the punctured saphenous vein under isoflurane anesthesia and glucose quantified using a glucometer (Contour next).

#### Data analysis

AO2P data were acquired at 1 Hz. Motion correction was based on the mRuby3 channel, and frames with the first derivative of the translation vector larger than 1 SD relative to baseline were excluded and the values linearly interpolated. Mean traces are median filtered over 60 s. The metric Δ*F*/*F*_0_ was calculated for each pixel relative to the average of the baseline 60 s before injections, and the average of a region of interest was taken from a region with minimal light absorption by superficial vasculature. Spatial maps of signal increase represent Δ*F*/*F*_0_ and are transparency weighted according at time point *F* (*F* is the average over the 1 min marked with yellow shade in Fig. 9 and *F*_0_ the 1 min before injections). All animal protocols were approved by the Institutional Animal Care and Use Committees at all participating institutions: Janelia Research Campus (25-0280), UC San Diego (S02174M), National Institute of Environmental Health Sciences (NIEHS) (2015-0013), University of Iowa (4092183).
