# Supplementary material for: iGlucoSnFR2: A genetically encoded fluorescent sensor for measuring intracellular or extracellular glucose in vivo in mouse brain
Source: Sci Adv. 2025 Nov 12;11(46):eadz3889. doi: 10.1126/sciadv.adz3889 (PMC12609159; doi:10.1126/sciadv.adz3889)
Supplement: Supplementary file 1 — Figs. S1 to S7 [file sciadv.adz3889_sm.pdf]

Science Advances
AAAS

Supplementary Materials for

iGlucoSnFR2: A genetically encoded fluorescent sensor for measuring intracellular or extracellular glucose in vivo in mouse brain

Jonathan S. Marvin *et al.*

Corresponding author: Jonathan S. Marvin, marvinj@janelia.hhmi.org

Sci. Adv. **11**, eadz3889 (2025)
DOI: 10.1126/sciadv.adz3889

This PDF file includes:

Figs. S1 to S7

Fig. S1.

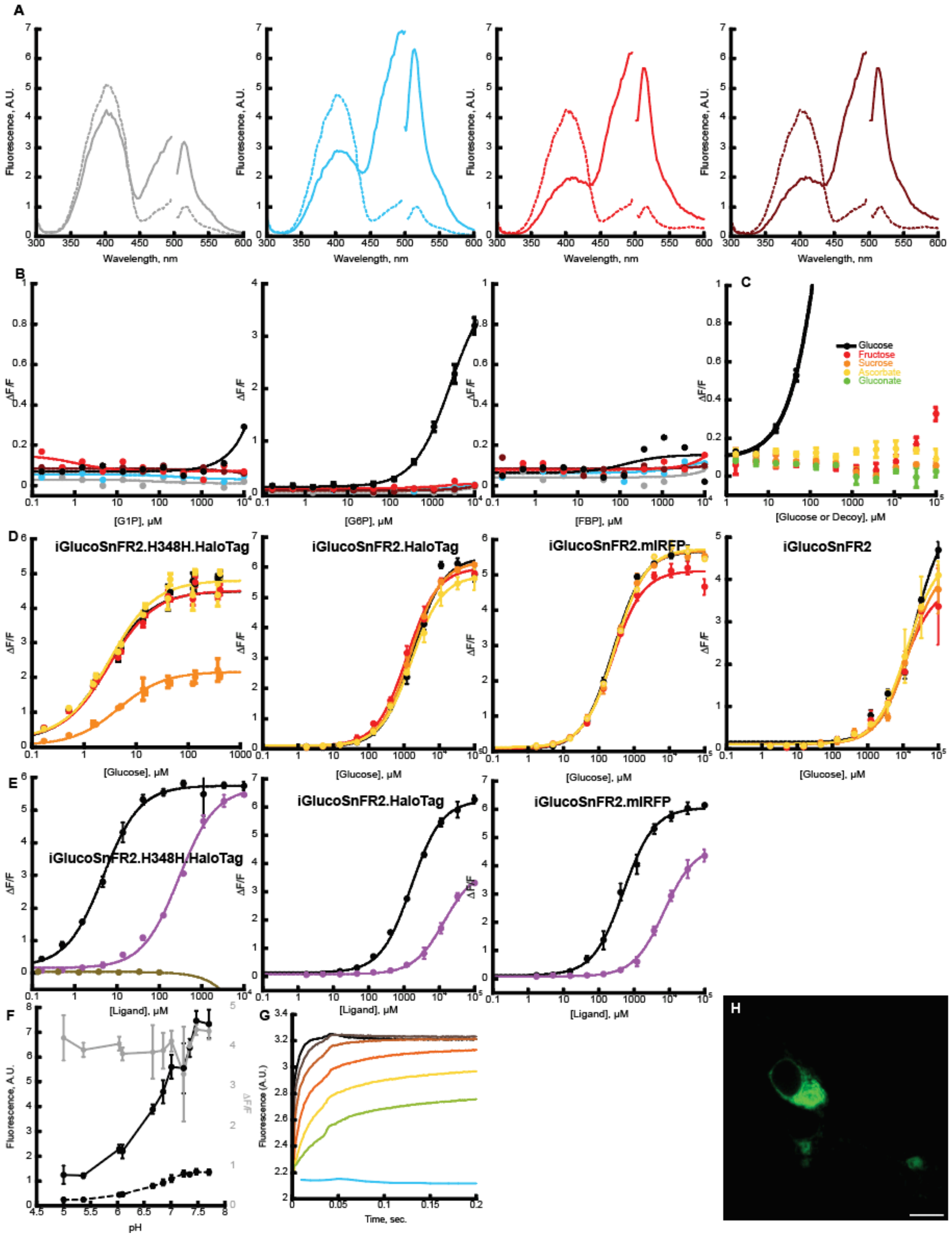

A
Fluorescence, A.U.
Wavelength, nm
B
C
ΔF/F
[G1P], μM
[G6P], μM
[FBP], μM
Glucose
Fructose
Sucrose
Ascorbate
Gluconate
[Glucose or Decoy], μM
D
iGlucoSnFR2.H348H.HaloTag
iGlucoSnFR2.HaloTag
iGlucoSnFR2.mIRFP
iGlucoSnFR2
[Glucose], μM
E
[Ligand], μM
F
pH
G
Fluorescence (A.U.)
Time, sec.
H

Fig. S1. *In Vitro* characterization of iGlucoSnFR2

(**A**) Excitation spectra (emission detected at 515 nm) and emission spectra (excitation at 485 nm) of iGlucoSnFR2 variants in the absence of glucose (dashed lines) and with 15 mM glucose (solid lines). 5 nm bandpass filters were used for all measurements. Grey, untagged sensor; blue, HaloTag; red, mRuby3; deep red, mIRFP670nano3. All plots normalized so that the emission intensity of the unliganded state is 1.

(**B**) Titration of iGlucoSnFR2 variants with phosphorylated decoys. Colours as above. Black, iGlucoSnFR2.H348H.HaloTag. The apparent millimolar affinity of H348H for G6P could be genuine, or contamination of the G6P compound with trace amounts of glucose.

(**C**) Titration of iGlucoSnFR2.mRuby3 with glucose or a panel of decoys. Black, glucose; red, fructose; orange, sucrose; yellow, ascorbate; green, gluconate.

(**D**) Titration of iGlucoSnFR2 variants (each graph is titled with the variant name) with glucose alone (black) and in the presence of 1 mM glucose-1-phosphate (red), glucose-6-phospate (orange), or fructose-1,6-bisphosphate (yellow). The apparent effect of G6P on the H348H (high affinity variant) is likely just contamination of the G6P stock with trace amounts of glucose.

(**E**) Titration of iGlucoSnFR2 variants (each graph titled with the variant name) with glucose (black) or 2-deoxyglucose (violet) or Glutor (brown).

(**F**) pH dependence of fluorescence for iGlucoSnFR2.HaloTag. In the presence of 15 mM glucose, black solid points and line. In the absence of glucose, black open points and dashed line. Difference between bound and unbound (ΔF/F), grey.

(**G**) Stopped flow fluorescence of iGlucoSnFR2.HaloTag binding glucose. Final concentrations of glucose are 100 mM (black), 33.33 mM (brown), 11.11 mM (deep red), 3.7 mM (orange), 1.23 mM (yellow), 0.4 mM (green), 0 mM (light blue).

(H) Confocal image (60x) of neurons transfected with AAV2/1.hSynapsin.(mem).iGlucoSnFR1. Scale bar 10 µm.

Fig. S2.

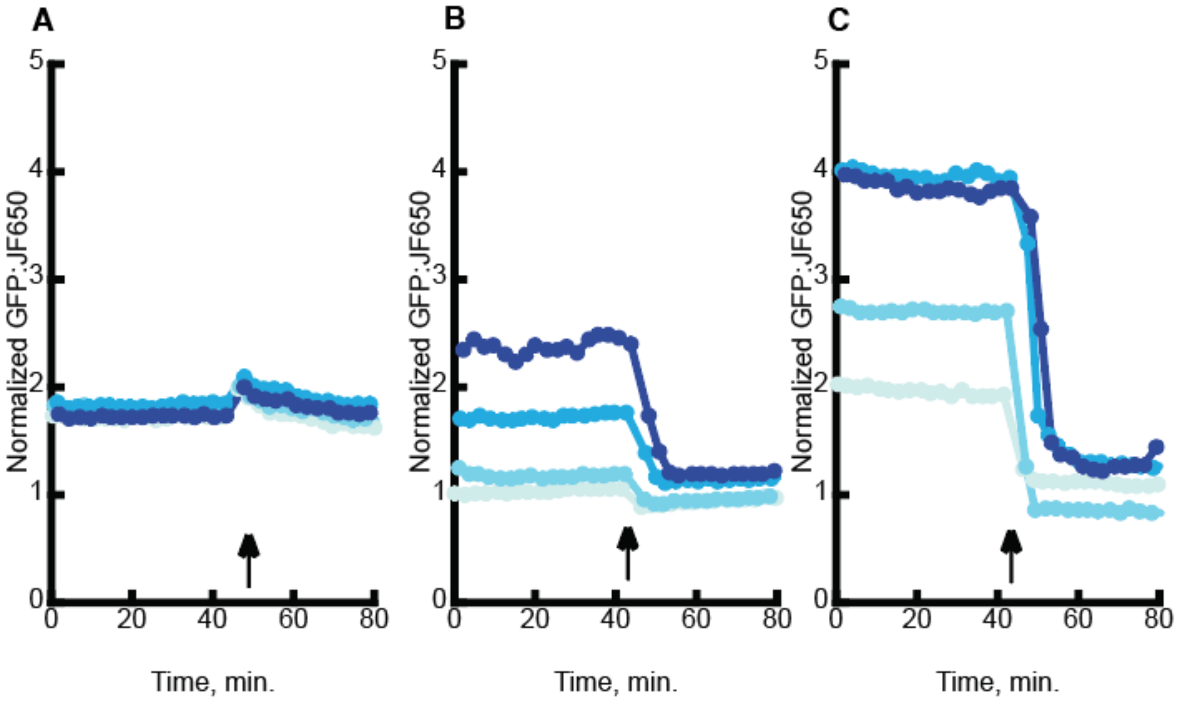

Fig. S2. Comparison of fluorescence response of iGlucoSnFR2 to iGlucoSnFR1 and cpSFGFP in HeLa cells. (**A**) (cyto).cpSFGFP, (**B**) (cyto).iGlucoSnFR1, (**C**) (cyto).iGlucoSnFR2 each with HaloTag-JFX650. Equilibration glucose concentration indicated by increasing blue hue: 1 mM, 2 mM, 5 mM, or 10 mM glucose. Arrow indicates time of treatment with 10 µM Glutor.

Fig. S3.

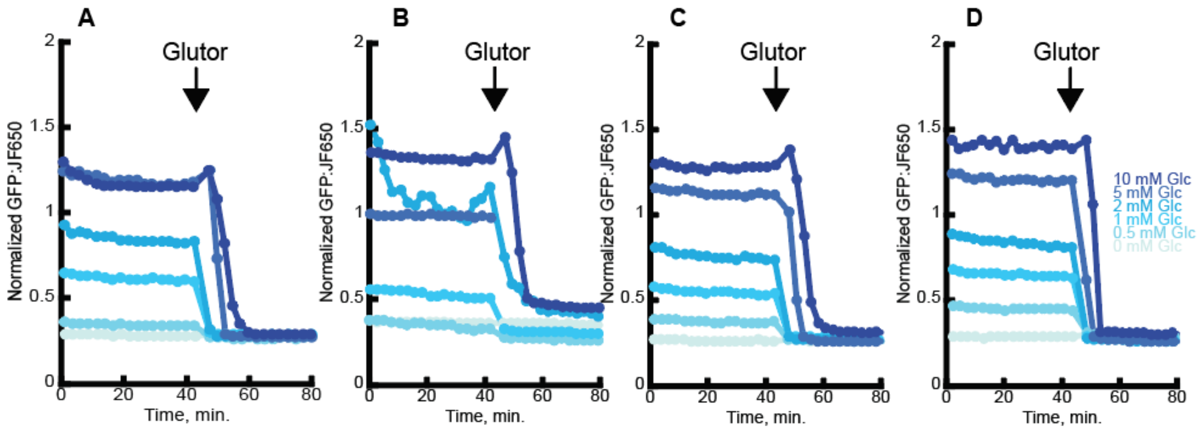

Fig. S3. Fluorescence response of (cyto).iGlucoSnFR2.HaloTag-JFX650 to Glutor treatment in different immortalized cell lines. (**A**) HeLa, (**B**) HEK293, (**C**) U2OS, (**D**) COS7. Equilibration glucose concentration indicated by increasing blue hue: 0 mM, 0.5 mM, 1 mM, 2 mM, 5 mM, or 10 mM glucose. Arrow indicates time of treatment with 10 µM Glutor.

Fig. S4.

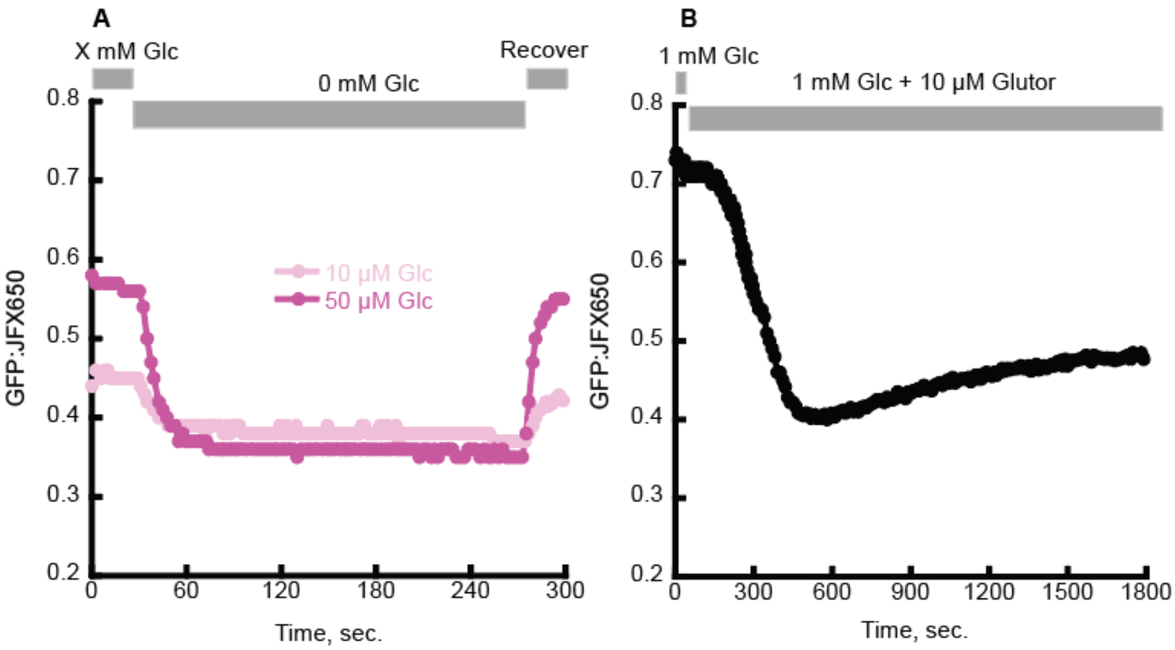

Fig. S4. Fluorescence response of (cyto).iGlucoSnFR2.H348H.HaloTag-JFX650 to varying concentrations of glucose perfusion or Glutor treatment. (**A**) Average fluorescence ratio of HeLa cells (N = 20) perfused with either 10 µM or 50 µM glucose, switched to 0 µM glucose, and then back to the starting concentration. (**B**) Average fluorescence ratio of those same cells after re-equilibration with 1 mM glucose and treated with 10 µM Glutor. Treatment periods are indicated by grey bars.

Fig. S5.

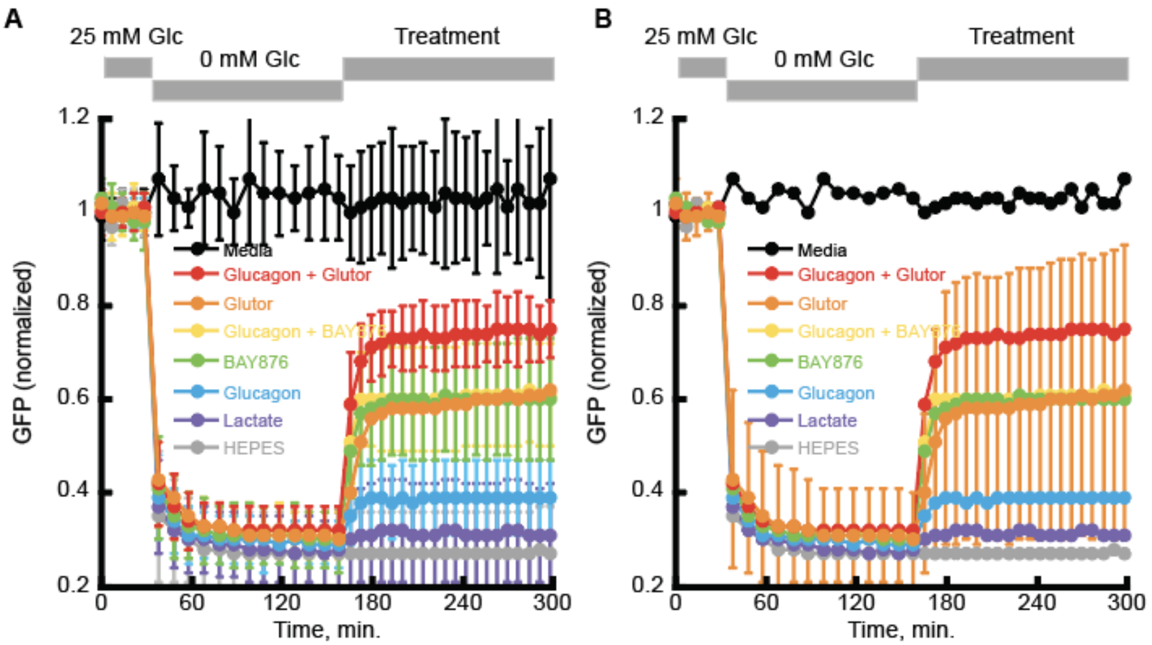

Fig. S5. iGlucoSnFR2 reports gluconeogenesis in hepatocytes in response to stimulating treatments and inhibition of glucose transport. Average normalized fluorescence of primary hepatocytes expressing (cyto).iGlucoSnFR2.mIRFP670nano3. Cells were imaged in primary culture media (with 25 mM glucose) for 30 minutes, then the media was replaced with HEPES buffer (0 mm glucose) for two hours. Gluconeogenic substrates (and glucose transport inhibitors) were added at the time indicated by the grey bars. Treatments were: No treatment (grey), 2 mM sodium pyruvate + 20 mM sodium lactate (purple), 2 mM sodium pyruvate + 100 nM Glucagon (blue), 2 mM sodium pyruvate + 5 µM BAY876 (green), 2 mM sodium pyruvate + 100 nM Glucagon + 5 µM BAY876 (yellow), 2 mM sodium pyruvate + 5 µM Glutor (orange), 2 mM sodium pyruvate + 100 nM Glucagon + 5 µM Glutor (red). Cells maintained in glucose rich media for the entirety are in black. Data are represented as mean of 60 cells. (**A**) The same data plotted in Fig. 5, but with the standard deviations included for all data points except Glutor treatment. (**B**) The same data plotted in Fig. 5, but with only the standard deviations for the Glutor treated cells (orange) plotted.

Fig. S6.

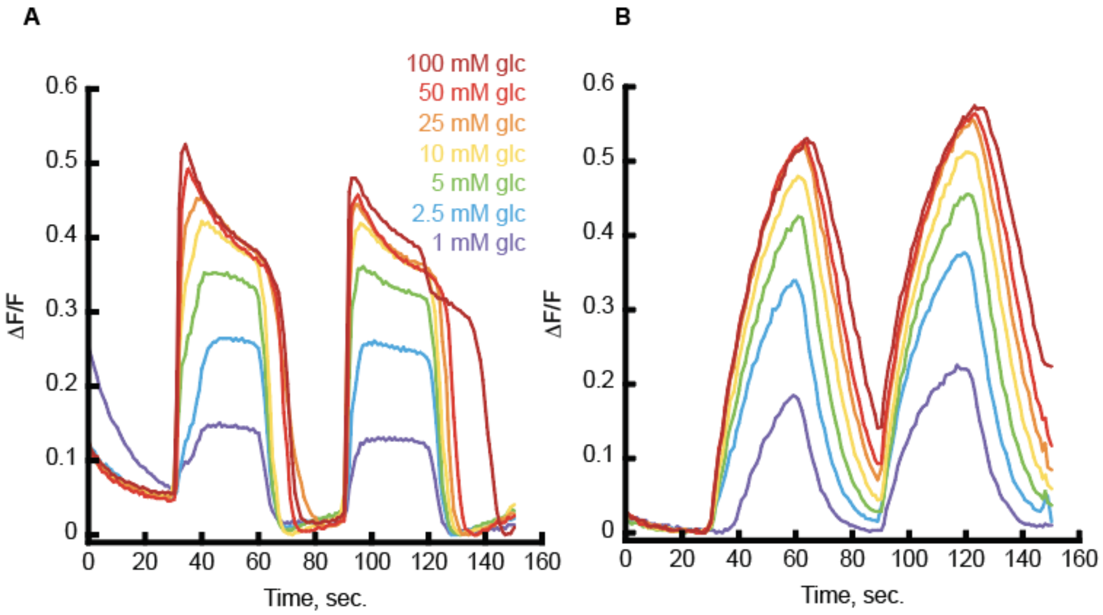

Fig. S6. Time of response to changes in extracellular glucose for iGlucoSnFR2. (A) Fluorescence response of cultured neurons transfected with pAAV.hSynap.(mem).iGlucoSnFR2 or (B) Response of pAAV.hSynap.(cyto).iGlucoSnFR2. Cultured neurons were continuously perfused with mammalian cell imaging buffer containing. Buffer was switched from 0 mM glucose to X mM glucose as indicated in the legend. Buffer was switched at 30 sec, 60 sec, 90, sec, and 120 sec. The rise in the fluorescence of the (mem).iGlucoSnFR2 during the first glucose perfusion is not as sharp as the rise in the second perfusion because the perfusion line retains some of the previous experiment's buffer. Average of 4 ROIs containing mostly neuronal processes (not cell bodies).

Fig. S7

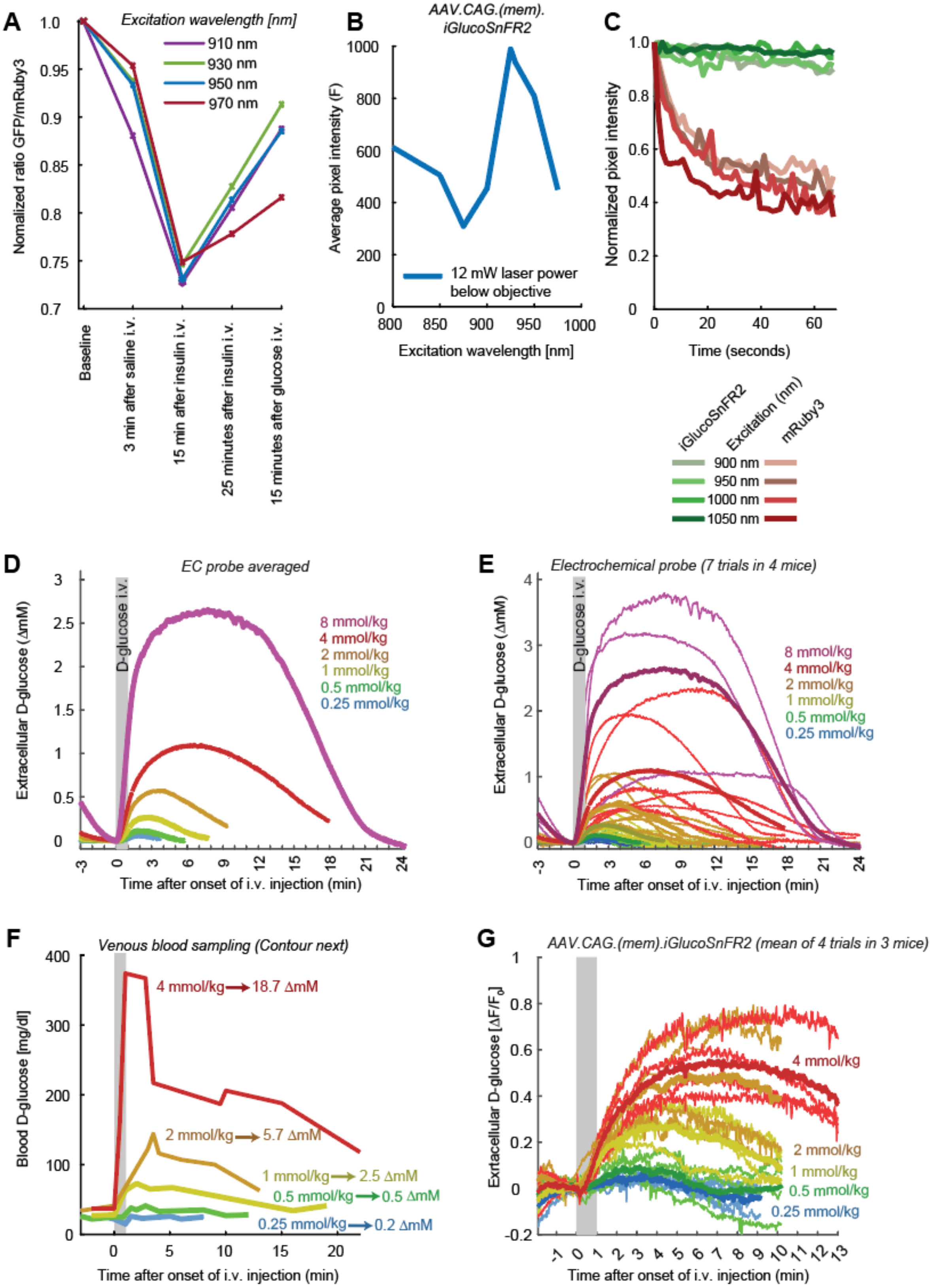

A
Excitation wavelength [nm]
910 nm
930 nm
950 nm
970 nm
Nomalized ratio GFP/mRuby3
Baseline
3 min after saline i.v.
15 min after insulin i.v.
25 minutes after insulin i.v.
15 minutes after glucose i.v.
B
AAV.CAG.(mem).
iGlucoSnFR2
Average pixel intensity (F)
12 mW laser power
below objective
Excitation wavelength [nm]
C
Normalized pixel intensity
Time (seconds)
iGlucoSnFR2
Excitation (nm)
mRuby3
900 nm
950 nm
1000 nm
1050 nm
D
EC probe averaged
D-glucose i.v.
Extracellular D-glucose (ΔmM)
8 mmol/kg
4 mmol/kg
2 mmol/kg
1 mmol/kg
0.5 mmol/kg
0.25 mmol/kg
Time after onset of i.v. injection (min)
E
Electrochemical probe (7 trials in 4 mice)
D-glucose i.v.
Extracellular D-glucose (ΔmM)
8 mmol/kg
4 mmol/kg
2 mmol/kg
1 mmol/kg
0.5 mmol/kg
0.25 mmol/kg
Time after onset of i.v. injection (min)
F
Venous blood sampling (Contour next)
Blood D-glucose [mg/dl]
4 mmol/kg → 18.7 ΔmM
2 mmol/kg → 5.7 ΔmM
1 mmol/kg → 2.5 ΔmM
0.5 mmol/kg → 0.5 ΔmM
0.25 mmol/kg → 0.2 ΔmM
Time after onset of i.v. injection (min)
G
AAV.CAG.(mem).iGlucoSnFR2 (mean of 4 trials in 3 mice)
Extracellular D-glucose [ΔF/F0]
4 mmol/kg
2 mmol/kg
1 mmol/kg
0.5 mmol/kg
0.25 mmol/kg
Time after onset of i.v. injection (min)

Fig. S7. Supporting data for *in vivo* iGlucoSnFR2 characterization with adaptive optics 2 photon microscopy.

(**A**) The mean iGlucoSnFR2 AO2P signal change (ΔF/F0) is indicated for different excitation wavelengths (910-970 nm) following saline, insulin and D-glucose i.v. injections.
(**B**) The brightness (A.U.) of iGlucoSnFR2 in AO2P using 12mW of excitation power (measured below objective) in green channel was measured for varying excitation wavelengths (800 to 980 nm).
(**C**) iGlucoSnFR2 showed significantly less photobleaching than simultaneously recorded mRuby3 for excitation wavelengths 900 nm to 1050 nm (each with 30 mW below objective).
(**D**) Exemplary recording of a calibrated electrochemical (EC) probe implanted in sV1 cortex with repeated injections of increasing dosages of D-glucose i.v. (0.25 to 8 mmol/kg) at low baseline blood glucose (post 2 IU/kg insulin i.v.).
(**E**) The full dataset includes 3 to 7 EC recordings per dosage in 4 individual mice. The bold trace indicates the mean trace for each dosage.
(**F**) Venous blood was sampled from the saphenous vein under isoflurane anesthesia during the tail vein D-glucose infusion protocol under insulin (2 IU/kg). The amplitude of glucose increase for each dosage is indicated in ΔmM.
(**G**) Individual trials (thin trace) and mean (thick trace) for full iGlucoSnFR2 dataset.
